# Multifaceted immune dysregulation characterizes individuals at-risk for rheumatoid arthritis

**DOI:** 10.1038/s41467-023-43091-8

**Published:** 2023-11-22

**Authors:** Eddie A. James, V. Michael Holers, Radhika Iyer, E. Barton Prideaux, Navin L. Rao, Cliff Rims, Virginia S. Muir, Sylvia E. Posso, Michelle S. Bloom, Amin Zia, Serra E. Elliott, Julia Z. Adamska, Rizi Ai, R. Camille Brewer, Jennifer A. Seifert, LauraKay Moss, Saman Barzideh, M. Kristen Demoruelle, Christopher C. Striebich, Yuko Okamoto, Enkhtsogt Sainbayar, Alexandra A. Crook, Ryan A. Peterson, Lauren A. Vanderlinden, Wei Wang, David L. Boyle, William H. Robinson, Jane H. Buckner, Gary S. Firestein, Kevin D. Deane

**Affiliations:** 1 Benaroya Research Institute, Seattle, WA 98101 USA; 2 Division of Rheumatology, University of Colorado Anschutz Medical Campus, Aurora, CO 80045 USA; 3 Division of Immunology and Rheumatology, Stanford University, Stanford, CA 94304 USA; 4 VA Palo Alto Health Care System, Palo Alto, CA 94550 USA; 5 Department of Chemistry and Biochemistry, University of California, San Diego, La Jolla, CA 92093 USA; 6 Janssen Research and Development, Spring House, PA 19477 USA; 7 Division of Rheumatology, Department of Internal Medicine, Tokyo Women’s Medical University School of Medicine, Tokyo, Japan; 8 Department of Biostatistics and Informatics, Colorado School of Public Health, University of Colorado Anschutz Medical Campus, Aurora, CO 80045 USA; 9 Department of Cellular and Molecular Medicine, University of California, San Diego, La Jolla, CA 92093 USA; 10 Division of Rheumatology, Allergy and Immunology, University of California, San Diego, La Jolla, CA 92093 USA

**Keywords:** Rheumatoid arthritis, Autoimmunity, Lymphocyte activation, Predictive markers

## Abstract

Molecular markers of autoimmunity, such as antibodies to citrullinated protein antigens (ACPA), are detectable prior to inflammatory arthritis (IA) in rheumatoid arthritis (RA) and may define a state that is ‘at-risk’ for future RA. Here we present a cross-sectional comparative analysis among three groups that include ACPA positive individuals without IA (At-Risk), ACPA negative individuals and individuals with early, ACPA positive clinical RA (Early RA). Differential methylation analysis among the groups identifies non-specific dysregulation in peripheral B, memory and naïve T cells in At-Risk participants, with more specific immunological pathway abnormalities in Early RA. Tetramer studies show increased abundance of T cells recognizing citrullinated (cit) epitopes in At-Risk participants, including expansion of T cells reactive to citrullinated cartilage intermediate layer protein I (cit-CILP); these T cells have Th1, Th17, and T stem cell memory-like phenotypes. Antibody-antigen array analyses show that antibodies targeting cit-clusterin, cit-fibrinogen and cit-histone H4 are elevated in At-Risk and Early RA participants, with the highest levels of antibodies detected in those with Early RA. These findings indicate that an ACPA positive at-risk state is associated with multifaceted immune dysregulation that may represent a potential opportunity for targeted intervention.

## Abstract (web summary)

The presence of antibodies to citrullinated protein antigens (ACPA) in peripheral blood represents a risk a state that is ‘at-risk’ for subsequent development of rheumatoid arthritis (RA). Here authors compare multiple molecular and immunological parameters in individuals who are ACPA positive without inflammatory arthritis, ACPA negative controls and patients diagnosed with ACPA positive early-stage RA to conclude that complex immunopathological processes are present in an ACPA positive state which may be targeted by future preventive approaches for RA.

## Introduction

Studies of seropositive rheumatoid arthritis (RA) show that markers of autoimmunity, including antibodies to citrullinated protein/peptide antigens (ACPA), rheumatoid factor (RF) and others can be present in blood many years prior to the clinically-apparent onset of inflammatory arthritis (IA) and classified RA [(reviewed in ref. ^1^)]. The period of autoantibody elevations before the onset of IA has been called ‘pre-RA’ and is reasonably viewed as a distinct stage toward development of clinically-apparent IA and classifiable RA^2^. Notably, ACPA(+) individuals, especially those with higher titers of the common clinically used ACPA assay ‘anti-cyclic citrullinated peptide antibody (anti-CCP)^3^, have a significantly elevated risk of developing future classified RA, and thus these individuals can be designated to be in an “At-Risk” state, and defined as such for studies^1,2,4–8^.

Importantly, the identification of a pre-RA period has underpinned the development and execution of several prevention trials in RA testing the ability of a variety of agents to prevent or delay the progression to IA and classified RA^9–13^. However, despite the increasing interest in disease prevention, major knowledge gaps exist regarding the causal pathways and pathogenic processes that promote the initial break in tolerance to citrullinated self-antigens in At-Risk populations. Some data suggest that epigenetic changes are present in such individuals^14^, but it is not known whether unique peripheral blood cell sub-populations exhibit distinct epigenetic changes in disease-related pathways. It has also been demonstrated that T cells in subjects with established RA recognize multiple citrullinated autoantigens^15^, and it has also been demonstrated that there may be abnormalities of T cell subsets in pre-RA^16^; however, it is not known how the antigen specificity and phenotypic profile of self-reactive T cells or B cells in pre-RA may differ from ACPA(−) individuals or individuals with classified RA. That is important given that previous studies have demonstrated that ACPA can emerge years prior to IA and increase over time in levels and numbers and types of citrullinated antigens being recognized^4,5,17–21^, strongly suggesting the associated presence of autoreactive T cells that provide help to drive these ACPA responses.

To address these questions, we assemble individuals who are ‘At-Risk’ for RA as defined by the presence of ACPA (in particular, anti-cyclic citrullinated peptide antibodies [anti-CCP3], a test with high specificity for RA and future RA^22–24^) as well as individuals with anti-CCP3 positive Early RA and anti-CCP3 negative controls and assess immunologic and epigenetic changes that are associated with the ACPA+ “at-risk” state. Differential methylation analysis identifies in the At-Risk individuals non-specific dysregulation within multiple cell subsets, while there are more specific immunological pathway abnormalities in Early RA. Tetramer studies show a specifically increased abundance of T cells that recognize citrullinated (cit) epitopes in At-Risk participants; in particular, in At-Risk participants there is an expansion of T cells reactive to citrullinated cartilage intermediate layer protein I (cit-CILP). Furthermore, these cit-reactive T cells exhibit Th1, Th17, and T stem cell memory-like phenotypes. Antibody-antigen array analyses show that antibodies targeting cit-clusterin, cit-fibrinogen and cit-histone H4 are elevated in At-Risk and are at higher levels in Early RA. In aggregate, these findings indicate that an ACPA positive at-risk state is associated with multifaceted immune dysregulation, which is likely to be related to the loss of self-tolerance to cit-antigens. These findings are relevant when considering emerging opportunities in RA prevention, as these pathways may represent potential therapeutic targets.

## Results

### Study participants

Full details of the study participants are included in the Methods section but, in brief, as part of the Targeting Immune Responses for Prevention of Rheumatoid Arthritis (TIP-RA) Collaborative we evaluated 97 participants who exhibited ACPA positivity by the commercial anti-CCP3 ELISA assay (IgG) (Inova Diagnostics, Inc., San Diego, CA; positive level >=20 units) who at their baseline study visit did not have historical or current examination evidence of IA; these participants are designated herein as “At-Risk”. In addition, 172 individuals without IA or anti-CCP3 positivity were evaluated; these participants are designated anti-CCP3(−) Controls. Finally, 62 individuals were enrolled who were anti-CCP3(+) with a clinical diagnosis of RA within 12 months of their study visit; these participants are designated “Early RA”.

There were no significant differences between At-Risk and anti-CCP3(−) Controls in age, gender, race, history of ever or current smoking, self-reported FDR status, BMI, the presence of >=1 allele containing the shared epitope, and positivity for high sensitivity C reactive protein (hsCRP) (Table 1). However, the At-Risk subjects had significantly higher levels of anti-CCP3, rheumatoid factor (RF) IgM and RF-IgA (Table 1). Between the At-Risk and Early RA groups there were no significant differences in gender, race, self-reported FDR status, BMI and presence of >=1 allele containing the shared epitope. However, in comparison to the At-Risk group, the Early RA participants were younger, and more were current smokers and had higher levels of anti-CCP3, RF-IgM and RF-IgA.

**Table 1:** Baseline characteristics of anti-CCP3(−) Controls, At-Risk and Early RA participants

	Anti-CCP3(−) Controls	At-Risk	Early RA	*P*-value anti-CCP3(−) to At-Risk*	*P*-value At-Risk to Early RA*
*n*	172	97	62	-	-
Age, mean (SD)	57.8 (12.6)	58.5 (12.6)	51.8 (12.9)	0.669	0.002
Sex, *n* (%) female	117 (68.0)	63 (64.9)	41 (66.1)	0.540	0.879
Race, *n* (%) NHW	135 (78.5)	79 (81.4)	43 (69.4)	0.562	0.081
BMI, mean (SD)	26.9 (5.8)	27.8 (4.9)	30.3 (6.1)	0.183	0.006
Smoking status					
*n* (%) ever smoked	59 (34.3)	35 (36.1)	33 (53.2)	0.769	0.033
*n* (%) currently smoking	5 (2.9)	6 (6.2)	15 (24.2)	0.054	0.005
Self-report FDR with RA, *n* (%)	48 (27.9)	18 (18.6)	10 (16.1)	0.082	0.694
Shared epitope present	81 (47.1)	49 (50.5)	40 (64.5)	0.590	0.081
*0401 present				0.351	0.122
0 alleles	139 (80.8)	71 (73.2)	38 (61.3)		
1 allele	32 (18.6)	25 (25.8)	23 (37.1)		
2 alleles	1 (0.6)	1 (1.0)	1 (1.6)		
Duration since initial identification of IA by a rheumatologist, mean number of days (SD)	-	-	55 (23)	-	-
Met ACR/EULAR 2010 criteria at baseline visit	-	-	61 (98.4)	-	-
CCP3 level, mean	4.1 (1.4)	82.9 (84.2)	210.5 (83.4)	**-**	**-**
CCP3 positivity >=20 units	0 (0.0)	97 (100.0)	62 (100.0)	**-**	**-**
RF-IgA level, mean	0.4 (1.4)	7.4 (20.5)	37.9 (42.7)	**-**	**-**
RF-IgA positivity				**-**	**-**
Low positive	2 (1.2)	0 (0.0)	2 (3.2)		
High positive (>3x ULN)	2 (1.2)	16 (16.5)	40 (64.5)		
RF-IgM level, mean	3.2 (9.8)	14.7 (30.4)	69.3 (46.2)	**-**	**-**
RF-IgM positivity				**-**	**-**
Low positive	15 (8.9)	9 (9.3)	3 (4.8)		
High positive (>3x ULN)	6 (3.5)	17 (17.5)	44 (71.0)		

Source data are provided as a Source Data file.

*CCP3* anti-cyclic citrullinated peptide 3 (IgG, Inova Diagnostics, Inc), *RA* rheumatoid arthritis, *SD* standard deviation, *NHW* non-Hispanic white, *BMI* body mass index, *FDR* first-degree relative, *IA* inflammatory arthritis, *ACR/EULAR* American College of Rheumatology/European League Against Rheumatism, *RF* rheumatoid factor, *Ig* immunoglobulin, *ULN* upper limit of normal.

*Comparisons were made using chi-squared testing for dichotomous variables, and *t* tests for continuous variables; a *p* < 0.05 was considered significant; two-sided testing was performed. We do not report *p*-values for comparison of autoantibody levels or positivity given ACPA was used as inclusion criteria.

### Differentially methylated loci and genes suggest uniquely expanded signaling pathways in lymphocyte subsets of At-Risk and Early RA individuals

One potential mechanism to drive the transition from an at-risk state to clinically-apparent IA and classifiable RA is through alterations in the epigenetic landscapes of T and B lymphocytes. Previous studies have revealed that the DNA methylation patterns in RA can be distinguished from controls in peripheral blood naïve T cell and peripheral blood mononuclear cells, including some published findings in ACPA(+) individuals without IA^14^. To extend these observations, we performed a cross-sectional analysis of DNA methylation signatures in sorted peripheral blood cells: B cells, memory T cells and naïve T cells.

**Fig. 1:**
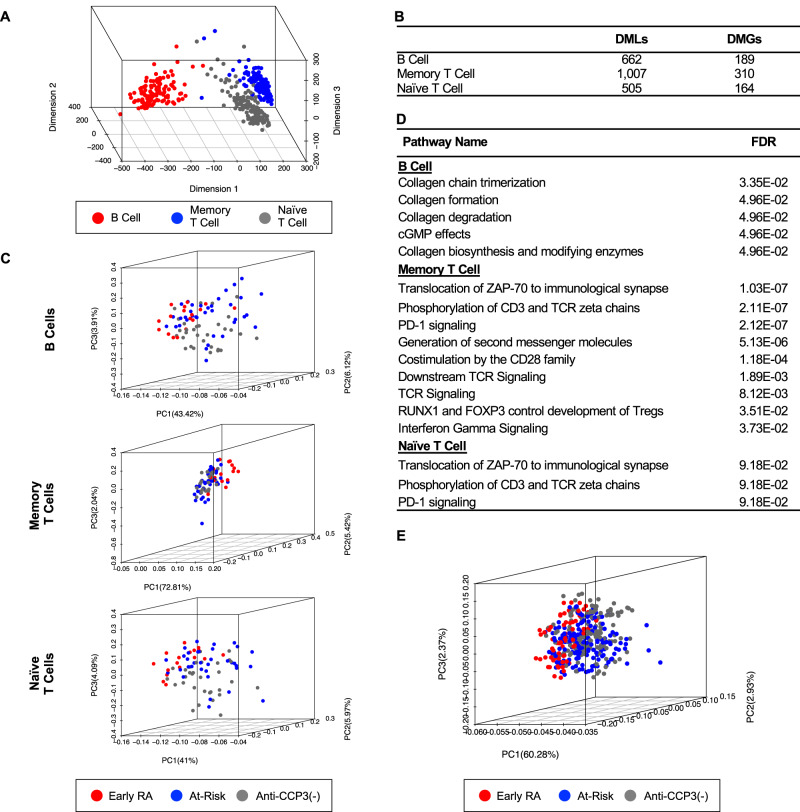
Methylation signatures of anti-CCP3(−), At-Risk and Early RA in peripheral blood B cells, memory T cells and naive T cells. **A** Dimension reduction analysis of samples on all filtered loci confirmed variances of methylation from cell lineage specific methylation in B cell, memory T cell and naїve T cells. **B** DML and DMG identified among anti-CCP3(−), At-Risk and Early RA in each cell type (Unadjusted *p* < 0.05, based on two-sided Student’s *t*-test and differences of average β > 0.1 were used as cutoffs for DML). **C** PCA of cohort 2 samples and DML on B cell, memory T cell and naїve T cells. **D** Selected differentially modified pathways identified in each cell lineage among anti-CCP3(−), At-Risk and Early RA samples. **E** Combined results of one-vs-one predictive models separates anti-CCP3(−) control, At-Risk and Early RA samples. DML differentially methylated locus, DMG differentially methylated genes, PCA principal component analysis. Source data are provided as a Source Data file.

Dimensional reduction analysis was performed on samples limited to females to eliminate the effects of sex-specific methylation in order to improve the power of the study to discriminate between the groups^25^. The data were evaluated in an initial sample set comparing all three groups called “cohort 1” (anti-CCP3(−) *n* = 21, At-Risk *n* = 20, Early RA *n* = 5) and a confirmatory “cohort 2” (anti-CCP3(−) *n* = 31, At-Risk *n* = 36, Early RA *n* = 17), with 789,935 filtered loci for initial validation (Fig. 1a). From the plot, samples were separated into the three lymphocyte lineages, with the first dimension distinguishing B cells from T cells and the second-dimension segregating memory T cells from naïve T cells. The dimension reduction analysis confirmed variance of methylation from cell lineage specific methylation in B cells, memory T cells and naïve T cells.

For cohort 1, 550, 294, and 404 differentially modified loci (DML) were identified among anti-CCP3(−), At-Risk and Early RA groups in B cells, memory T cells and naïve T cells, respectively (Supplementary Data 1). For cohort 2, 124, 734, and 121 DML were found in corresponding cell populations. PCA plots (Fig. 1c) showed separation of anti-CCP3(−), At-Risk and Early RA groups in each of the three lymphocyte types by DML in confirmatory cohort. Notably, hierarchical clustering confirmed separation in the 3 groups although the visual differences were more subtle than in PCA as expected due to the significant heterogeneity found within our dataset (Supplementary Fig. 1). The DML overlap between the two cohorts was highly significant (*p* = 1.71 × 10^−22^, 8.17 × 10^−33^ and 3.13 × 10^−44^ in B cell, memory T cell and Naïve T cells, respectively, by hypergeometric tests). This concordance allowed for combination of the DML for both the initial and confirmatory sample sets (Fig. 1b) and shows that batch effects were minimal.

*Comparison of anti-CCP3(−), At-Risk and Early RA participants* The union of DML across cohorts 1 and 2 corresponded to 662, 1007, and 505 DML in B cells, memory T cells and naïve T cells, respectively, across anti-CCP3(−) Controls, At-Risk and Early RA groups. Genes with DML located in the promoter or gene body were defined as DMG and 198, 310, and 164 DMG were identified in B cells, memory T cells and naïve T cells, respectively. Pathway enrichment analysis based on these DMG identified differentially methylated pathways where significant differences were observed by comparing anti-CCP3(−) Controls, At-Risk and Early RA participants for each lineage with the most significant differences appearing in memory T cells and B cells (FDR < 0.1, see Fig. 1d and Supplementary Table 1). Memory T cell pathways are highly relevant to T cell signaling and adaptive immune responses. For example, memory T cell pathways included “Interferon gamma signaling” (FDR = 3.73 × 10^−02^), “Translocation of ZAP-70 to Immunological synapse” (FDR = 1.03 × 10^−07^), and “Phosphorylation of CD3 and TCR zeta chains” (FDR = 2.11 × 10^−07^) and “PD-1 signaling” (FDR = 2.12 × 10^−07^). B cell pathways are focused on organization of the extracellular matrix (“Collagen chain trimerization”, FDR = 3.35 × 10−^02^; “Collagen formation”, FDR = 4.96 × 10^−^^02^; “Collagen degradation”, FDR = 4.69 × 10^−02^; and “Collagen biosynthesis and modifying enzymes”, 4.96 × 10^−02^).

*Classification model of anti-CCP3(−), At-Risk and Early RA participants* A random forest classification approach was used to explore key features and distinguish between anti-CCP3(−), At-Risk and Early RA participants. Pooled samples from cohorts 1 and 2 (anti-CCP3(−) Controls = 52 participants, 152 samples; At-Risk = 56 participants, 164 samples; and Early RA = 22 participants, 65 samples) were first split into training and test data sets. One-vs-one random forest classification models were then trained on the previously identified DML using 10-fold cross validation. After feature selection, the models achieved accuracies of 89.7%, 78.1%, and 96.8% on the test data when discriminating between Early RA vs At-Risk, Early RA vs anti-CCP3(−) Controls and At-Risk vs anti-CCP3(−) Controls, respectively. Principal components analysis using the top 10% of features, by importance, combined from each one-vs-one model distinguished between samples by RA status (Fig. 1e and Supplementary Data 2).

*Pairwise group comparisons of anti-CCP3(−), At-Risk and Early RA participants* In the previous analysis, all three groups were compared to determine the global differences. We then performed pairwise comparisons between the individual groups to help determine the primary source of the group differences. We identified 86, 77 and 83 DML associated with 25, 22, and 28 DMG between anti-CCP3(−) and At-Risk participants in B cells, memory T cells and naïve T cells, respectively, but only a single enriched pathway was identified in the pairwise analysis (*p* < 0.01). However, in comparisons between At-Risk and Early RA participants, we identified 359, 278, and 270 DML in B cells, memory T cells and naïve T cells, respectively, that mapped to 112, 82, and 101 DMG. Comparison of At-Risk and Early RA participants showed multiple enriched pathways within Early RA related to T cell signaling (*p* < 0.01). These data suggest that progression from At-Risk to Early RA and involves remodeling the methylome at key genes related to T cell function.

In aggregate, these findings of DNA methylation patterns demonstrate that there is ‘general’ immune dysregulation in At-Risk compared to anti-CCP3(−) Controls, but more defined abnormalities between Early RA and At-Risk that may might identify pathways that are key to a transition to RA, and potentially be targets for prevention or early treatment. Furthermore, the classification model and selected DML discriminate among anti-CCP3(−) Controls, At-Risk and Early RA participants, and thus could ultimately lead to predictive algorithms that identify individuals at high-risk for progression to RA.

### Expanded cartilage intermediate layer protein (CILP)-specific and phenotypically altered T lymphocytes in At-Risk individuals

Given the differential methylome signature found across B and T lymphocyte populations, another potential mechanism to account for the activation of autoreactive B cells and development of serum ACPA in At-Risk individuals is a dysregulated expansion of antigen-specific T cells that recognize citrullinated self-peptides^15,26,27^. To address this question, we utilized HLA Class II tetramers (Tmr) to determine the frequency and phenotype of T cells specific for citrullinated antigens directly from PBMC using a multiplex staining approach^28^. We assessed the frequency of DRB1*04:01 restricted T cells specific for citrullinated epitopes  to which immune responses were previously shown to be present in patients with established RA, including citrullinated (cit) forms of aggrecan^29^, CILP (isoform 1)^15^, vimentin and fibrinogen^15^, and α-enolase^30^, as well as T cells specific for an influenza peptide as an experimental control. For these studies, we evaluated anti-CCP3(−) Controls, At-Risk, and Early RA participants who had DRB1*04:01 haplotypes (Supplementary Fig. 2).

**Fig. 2:**
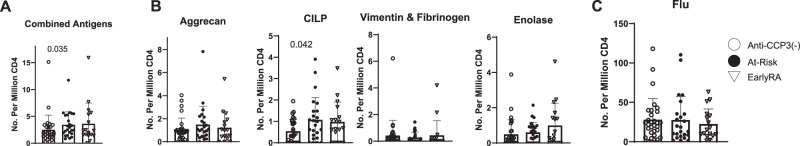
The frequency of cit-reactive T cells is increased in At-Risk subjects. Samples from the subset of Anti-CCP3(−), At-Risk, and Early RA participants who were DRB1*04:01 positive (30, 24, and 17 subjects, respectively) were stained with HLA class II tetramers to enumerate CD4 + T cells specific for citrullinated (cit) aggrecan, cartilage intermediate layer protein (CILP), vimentin and fibrinogen, or α-enolase. **A** The combined frequency of cit-reactive T cells was significantly higher in At-Risk participants compared to Anti-CCP3(−) controls (*p* = 0.035), but was not significantly different between At-Risk and Early RA participants. **B** Considering individual antigen specificities, the frequency of CILP reactive T cells was significantly higher for At-Risk subjects compared to Anti-CCP3(−) Controls and Early RA participants (*p* = 0.042). Only modest differences were seen for other antigens, indicating that the increase in cit-specific T cells in At-Risk subjects was driven by CILP reactive T cells. **C** The frequency of influenza (flu) reactive T cells did not differ between Anti-CCP3(−) Controls, At-Risk, and Early RA participants. Error bars indicate standard deviation. *P*-values indicate unpaired comparisons using Wilcoxon’s nonparametric two-tailed test. Source data are provided as a Source Data file.

We found At-Risk participants to have a significantly higher frequency of cit-specific T cells than anti-CCP3(−) Controls; in addition, the frequency of cit-specific T cells was similar between At-Risk and Early RA participants (Fig. 2a). This overall increase in cit-specific T cells in the At-Risk participants as compared to the anti-CCP3(−) Controls was primarily driven by increased numbers of cit-CILP-specific T cells, whereas the other specificities contributed more modestly to the observed increase (Fig. 2b). In contrast, the frequency of influenza specific T cells did not differ between anti-CCP3(−), At-Risk and Early RA participants (Fig. 2c).

**Fig. 3:**
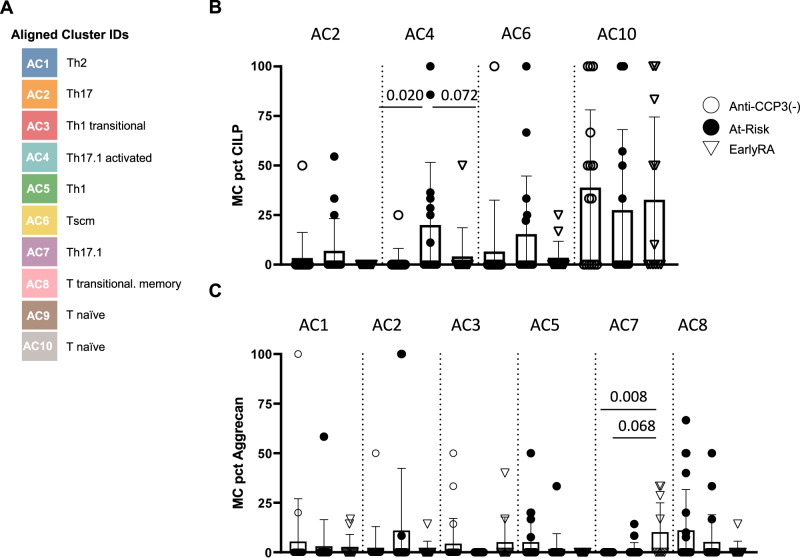
Examining the phenotypes of CILP specific CD4 + T cells. **A** Aligned clusters within the CD4 + T cell landscape were classified into T cell surface phenotype groups based on six surface markers (CD45RA, CD38, CCR7, CXCR3, CCR4, and CCD6) using the DISCOV-R approach and assigned identities as elaborated in Supplementary Fig. 3. **B** For At-Risk participants, a significantly higher proportion of cartilage intermediate layer protein (CILP) reactive T cells resided within AC4 as compared with Anti-CCP3(−) Controls (*p* = 0.020). Early RA participants also tended to have a lower proportion of CILP reactive T cells within AC4 (*p* = 0.072). **C** For Early RA participants a significantly lower proportion of aggrecan reactive T cells resided within AC7 as compared with Anti-CCP3(−) Controls (*p* = 0.008). Early RA participants also tended to have a higher proportion of aggrecan reactive T cells within AC7 (*p* = 0.06). Those with at least 8 total antigen specific events are shown. For CILP graph anti-CCP3(−) *N* = 15, at-risk *N* = 16, and early-RA *N* = 12. For aggrecan graph anti-CCP3(−) *N* = 22, at-risk *N* = 19, early-RA *N* = 11. Error bars indicate standard deviation. *P*-values indicate unpaired comparisons using Wilcoxon’s nonparametric two-tailed test. Source data are provided as a Source Data file.

We further hypothesized that the expansion of self-reactive T cells would be accompanied by the acquisition of specific disease-associated T cell phenotypes in these cells. Therefore, we next investigated the surface phenotypes of cit-specific CD4 + T cells. This was accomplished by first defining an overall landscape for CD4 + T cells based on six phenotypic markers included in the flow cytometry panel (CD45RA, CD38, CCR4, CCR6, CXCR3, and CCR7), performing multidimensional clustering with DISCOV-R, and then overlaying Tmr+ cells onto that landscape^31^. This analysis yielded 10 distinct aligned clusters (Supplementary Fig. 3), which could be assigned to aligned cluster (AC) phenotypes based on specific combinations of surface marker expression (Fig. 3a and Supplementary Fig. 3). Among the different citrullinated epitope groups, only cit-CILP-specific and cit-aggrecan specific T cells exhibited significant differences in phenotype between participant groups. Notably, the At-Risk participants had increased proportions of CILP-specific cells in a Th17.1 cluster (AC4) when compared to anti-CCP3(−) controls (Fig. 3b). The Early RA participants had increased proportions of aggrecan-specific cells in another Th17.1 cluster (AC7) when compared to anti-CCP3(−) Controls (Fig. 3c). In contrast, influenza specific T cells were predominantly found to occupy the Th1 *AC5 and Th17.1 *AC4 clusters in all participant groups (Supplementary Fig. 4).

In assessments of the global CD4 + T cell responses with respect to their distribution across the aligned clusters defined by DISCOV-R, we observed a higher proportion of Th2-like cells (AC1) and a lower proportion of Th17 cells (AC2) and Th17.1 cells (AC4) in Early RA participants compared to anti-CCP3(−) (Supplementary Fig. 5). We also observed that a higher proportion of TSCM-like cells (AC6) was present in At-Risk participants compared to Early RA, and that a lower proportion of cells within a transitional Th1 cluster (AC3) was present in At-Risk participants compared to anti-CCP3(−) Controls or Early RA. These observations reveal apparent changes in the polarization of non-disease related T cells. Further, there was a notable contrast between the global differences seen for total CD4 + T cells (a lower proportion of Th17.1 cells in At-risk and Early RA than in anti-CCP3(−) Controls) and the differences seen for cit-CILP (a higher proportion of Th17.1 cells in at-risk) and cit-Agg (a higher proportion of Th17.1 cells Early RA than in anti-CCP3(−) Controls).

In aggregate, these findings provide evidence that T cells specific to citrullinated antigens are expanded in ACPA+ At-Risk individuals. In particular, of the antigens evaluated herein, changes in the frequency of T cells specific for cit-CILP are predominant in the At-Risk participants. Moreover, the phenotype of cit-CILP-specific T cells is distinct in the At-Risk participants (as compared with other participants) and displayed surface markers associated with pro-inflammatory features, supporting a role for these cells and inflammatory pathways in the initial break in tolerance and potentially to further progression to clinical disease.

### Multiple serum antibody levels to citrullinated and non-citrullinated antigens are elevated in Early RA compared to At-Risk, with limited but informative differences between At-Risk and anti-CCP3(−) Controls

We profiled the serum antibody reactivity to 41 citrullinated and non-citrullinated proteins and peptides (including CILP) on a multiplex antigen bead array in 172 anti-CCP(−) Controls, 97 At-Risk and 62 Early RA participants. A list of the antigens in the assay is provided in Supplementary Data 3.

We performed pairwise comparisons of antibodies as levels to individual antigens as well as means of summed levels of antibodies to groups of similar antigens (e.g., levels of antibodies to all fibrinogen-containing antigens) between At-Risk and anti-CCP3(−) Controls, and At-Risk and Early RA participants in analyses that were adjusted for age, sex and smoking status (Supplementary Data 3). In these analyses, there was a significant increase in a summed level of antibodies to citrullinated (cit) clusterin-related antigens in At-Risk participants (*p* = 0.044), although with a stricter multiple testing false-discovery rate (FDR)-adjusted *p*-value (0.05 criteria), this finding was not significant. However, no other antibody level to a specific antigen, or summed antigens, was significantly different between the At-Risk and anti-CCP3(−) Control participants – including antibodies to CILP (citrullinated or non-citrullinated). In contrast, in comparisons between Early RA and At-Risk participants, levels of multiple antibodies to individual antigens, or groups of antigens (and including individual and summed antibody levels to citrullinated CILP) were significantly elevated in Early RA using a *p*-value of 0.05 as well as the stricter FDR-adjusted *p*-value.

We also performed pairwise comparisons of each antibody as “positive” or “negative” based on a level that was >= 3 standard deviations above the mean level in the anti-CCP3(−) Controls; furthermore, we evaluated total ‘counts’ of autoantibodies that were positive including by groups of similar antigens (e.g., cit-clusterin) (Supplementary Fig. 6 and Supplementary Data 4). In these analyses, compared to anti-CCP3(−) Controls, At-Risk participants had a higher prevalence of positivity at a *p* < 0.05 level of antibodies to multiple individual antigens including forms of cit-fibrinogen, cit-clusterin, cit-fillagrin and cit-histone H4 [H4 33-48 cit 39-40 (79)], as well as higher total counts of overall autoantibody positivity as well as summed antibody positivity to cit-clusterin, cit-fibrinogen and cit-histone H4 antigens. However, of these, only positivity to citrullinated histone H4 as a single antigen, and summed levels of total antibody positivity as well as to summed antibody positivity to cit-clusterin, cit-fibrinogen and cit-Histone H4 antigens were significant using the stricter FDR-adjusted *p*-value. In contrast, in comparisons between Early RA and At-Risk participants, the prevalence of positivity of multiple antibodies to individual antigens were significantly elevated in Early RA using a *p*-value of 0.05 as well as the stricter FDR-adjusted *p*-values. Notably, while there were significant elevations of antibodies to multiple non-citrullinated antigens in Early RA compared to At-Risk participants, the reactivities to citrullinated forms of the antigens were predominant.

To compare with the aforementioned Tmr analyses that were performed only in individuals who were positive at least one allele containing *0401, we further evaluated antibody levels within participants who were stratified by the presence or absence of *0401. In these analyses, in pairwise comparisons of the *0401 negative At-Risk and anti-CCP3(−) participants at the *p* < 0.05 level there was a significantly higher level of the summed level of antibodies to cit-clusterin antigens in At-Risk compared to anti-CCP3(−) subjects (*p* = 0.033) as well as to the individual autoantibody to cit-clusterin 231–250 (*p* = 0.046) (Supplementary Fig. 7 and Supplementary Data 5); however, the significance was lost using the stricter FDR-adjusted *p*-value. In the *0401 positive participants, there was also a higher summed level of antibodies to cit-clusterin antigens in the At-Risk subjects compared to the anti-CCP3(−) controls, although this was not statistically significant even at the *p* < 0.05 level. In contrast, in pairwise comparisons of Early RA and At-Risk participants (and with or without *0401 positivity), levels of antibodies to a number of antigens, including citrullinated CILP, were significantly elevated in Early RA at both the *p* < 0.05 and FDR-adjusted *p*-value levels. Notably, reactivities were overall similar between *0401 positive and negative participants (Supplementary Data 5); in addition, there were no significant correlations between levels of T cells reactive to CILP antigens and levels of anti-CILP antibodies (Supplementary Fig. 7).

We also performed pairwise comparisons of each antibody as “positive” or “negative” with participants stratified by *0401 status (Supplementary Figs. 8 and 9, and Supplementary Data 6). In these analyses, when compared to *0401 negative anti-CCP3(−) Controls, *0401 negative At-Risk participants had a higher prevalence of positivity at a *p* < 0.05 level of antibodies to multiple individual antigens including forms of cit-fibrinogen, cit-clusterin, and cit-histone H4; in addition, the At-Risk participants had higher total counts of overall autoantibody positivity as well as summed antibody positivity to cit-clusterin, cit-fibrinogen and cit-histone H4 antigens (Supplementary Figs. 8 and 9, and Supplementary Data 6). However, only total antibody positivity, and antibody positivity to summed cit-clusterin, cit-fibrinogen and cit-histone H4 remained significant at the FDR-adjusted *p*-value. Furthermore, still within the *0401 negative participants, there were multiple antibodies to individual antigens or groups of antigens that were elevated within Early RA participants compared to At-Risk. Finally, within the *0401 positive participants, there were no significant differences between anti-CCP3(−) Controls and At-Risk participants in any antibody positivity although there were multiple positive antibodies to individual antigens, or groups of antigens, in Early RA participants compared to At-Risk at both the *p* < 0.05 and FDR-adjusted *p*-value levels.

In aggregate, these findings demonstrate that compared to anti-CCP3(−) Controls, At-Risk participants have significant increases in antibody levels and/or positivity to a limited number of antigens including cit-clusterin, fibrinogen, fillagrin, with the most statistically robust finding of increased positivity to the individual antigen of cit-histone H4 and summed positivity of total antibody and combined cit-clusterin, cit-fibrinogen and cit-histone H4. In contrast, there are elevations and positivity of multiple antibodies, including antibodies to citrullinated CILP, in Early RA compared to At-Risk participants. This suggests that expanded antibody levels/positivity may be an integral part of a transition from an At-Risk state to clinically-apparent RA. Furthermore, the finding that CILP-reactive T cells are elevated in At-Risk participants in absence of significant elevations of anti-CILP antibodies suggests that for this antigen, T cell reactivity that is present within circulating PBMC may precede certain autoantibody elevations. Longitudinal studies should help to clarify these progressive changes over time.

## Discussion

Understanding the key molecular and other informative phenotypic differences that characterize At-Risk individuals from control and classified RA populations is a necessary step toward the design and implementation of mechanism-based prevention trials, including using drugs that may not be effective in treating patients with classified RA who have developed synovitis. In these studies, we have interrogated the epigenetic landscape, antigen-specific T cells and their phenotypes and autoantibody profiles. While we have not yet performed longitudinal analyses, the results presented herein identify a number of important points.

First, we have identified broad methylation signatures that differentiate At-Risk, Early RA and anti-CCP3(−) populations in peripheral blood B cells, memory T cells and naïve T cells. Notably, the methylome of Early RA demonstrated a pattern distinct from At-Risk individuals, indicating that progression to RA is accompanied by epigenetic remodeling, especially in T cell signaling. Interestingly, only a single pathway was found between anti-CCP3(−) controls and At-Risk despite a large number of DML. These data suggest that the methylome changes at the earliest stages of pre-RA might be random and that the accumulation of epigenetic marks in immunological pathways is a feature of RA transition. Supporting the latter notion, previous studies have documented increased serum levels of various cytokines during pre-RA without a dominant initial factor identified^17^, and that some of these cytokines can regulate DNA methyltransferases in RA synovial cells^32^. However, the progression towards disease involves remodeling the methylome in a more focused fashion and targets loci related to immune function. These signatures are likely to represent pathways that are critical to transitions from ACPA(+) state to clinically-apparent IA and classifiable RA; furthermore, these pathways could be targeted by interventions aimed to prevent this critical transition. In addition, further understanding of the epigenetic differences between At-Risk and anti-CCP3(−) controls, and integration with emerging data suggesting the presence of different endotypes in the pre-RA period should inform regarding the various pathways^18^, perhaps driven by unique mucosal/systemic immune site interactions or the differential influence of environmental factors on these early steps^33^. Age can potentially affect DNA methylation, and the Early RA group was slightly younger than the at-Risk and control cohorts. However, the mean ages of three groups were all within 5 years of each other within the 6^th^ decade of life. The rate of smoking was also modestly greater for Early RA. An effect of smoking on the blood DNA methylome has been described, although it is modest and does not involve the immunological pathways that we identified^34^.

Our tetramer-based analysis of frequency of T cells specific for citrullinated antigens indicated an expansion of cit-specific T cells in At-Risk individuals. Notably, this observed increase was driven by cit-CILP-reactive T cells, an antigen that was not previously recognized as a prominent T cell target in established RA^15^. This raises the possibility that the hierarchy of self-antigens targeted by T cells may change at different stages of disease development. Of note, CILP has been primarily described as a joint-based protein and been implicated in osteoarthritis and spinal disc disease, and antibody to non-citrullinated CILP as well as T cell reactivity to cit-CILP have been demonstrated in patients with classified RA^15,35^. However, CILP expression has also been described in cardiac fibroblasts in response to pulmonary hypertension, and it is known to be expressed in murine lung tissue^36^ although its presence in other non-articular tissue is not well described. Therefore, the broader role of autoimmunity to this protein in pre-RA and a transition to RA, including immune responses driven by non-articular antigen, will need to be explored in future studies.

There was no further increase in the frequency of cit-specific T cells in the Early RA group, but it could be speculated that additional self-reactive T cells may then be sequestered within affected joints. Alternatively, new T cell specificities (such as citrullinated histone H4, or clusterin, which has not been reported as a T cell antigen) may arise as At-Risk individuals progress to develop classifiable disease. Tetramer-based analysis of the phenotype of T cells specific for citrullinated antigens also provided several important insights. In general, cit-reactive T cells resided within a broad range of phenotypic clusters, implying that these self-reactive T cells can take on a broad range of functional phenotypes. Notably, both At-Risk and Early RA subjects had increased proportions of Th17.1-like T cells, albeit with different specificities and different cluster designations in At-Risk versus Early RA. This parallel enrichment appears to suggest a role for Th17.1 T cells (typically thought to secrete IL-17 and interferon γ) both in the initial break in tolerance and further disease progression. Minor deviations in the phenotype of total CD4 + T cells were also observed, indicating more global dysregulation of T cells. These observations harmonize with the observation of DML related to memory T cell function in particular.

The results of the antibody array testing are intriguing. While the At-Risk and anti-CCP3(−) individuals are clearly differentiated by elevated/positive levels of the commercially-available anti-CCP3 assay, in the antibody array utilized herein, there were elevations/positivity in the At-Risk group compared to the anti-CCP3(−) controls in a limited number of antibody-antigen sets including cit-clusterin, cit-fibrinogen, cit-fillagrin and cit-histone H4. In contrast, there were a large number of significant autoantibody elevations and positivity in the Early RA participants compared to the At-Risk. These findings are similar to those from other cross-sectional studies where there were limited elevations/positivity of ACPA fine specificities in at-risk individuals compared to individuals with classified RA^21^. Furthermore, because the approaches used herein to identify the At-Risk group included population-based screening (e.g., health-fairs and FDR testing) of individuals who had not sought health-care for joint symptoms may have created a group that is at a very early stage of pre-RA prior to significant expansion of many autoantibodies, especially if the antigens used are primarily those that are targeted in a stage of RA development when articular inflammation is prominent. Moreover, the antigens contained on the anti-CCP3 assay are proprietary and unknown to the research community. As such, a possible explanation for the discrepancy between anti-CCP3 levels and the ACPA array may be that the commercial assay is detecting a different set of antigens that is contained on the ACPA array that was developed based largely on antigens contained within the inflamed synovium in RA^37^. To address these issues, future studies should evaluate antibodies responses to alternative antigens that may be more relevant to breaks-in-tolerance that are specific for the pre-RA period, as well as to prospectively follow the evolution of antibodies through to the development of clinically-apparent IA to identify potentially key antibody-antigen systems that drive the transition to clinically-apparent disease. This latter point is especially important because published longitudinal studies have prospectively identified epitope spreading of ACPA prior to the onset of clinically-apparent IA^19,20^.

Notably, elevations of antibodies to cit-clusterin and cit-histone H4 were prominent herein in At-Risk participants compared to anti-CCP3(−) controls. Clusterin is a molecular chaperone that has been described as elevated in the circulation in early RA and associated with increased disease activity^38^; in addition, antibodies to cit-clusterin have been noted to be elevated in pre-RA in individuals who later developed clinically-apparent RA^39^. Clusterin is also expressed by mononuclear cells at sites of mucosal inflammation in diseases such as Crohn’s^40^ and it has been demonstrated to play a role in modulation of lung inflammation^41^. Therefore, it may be that antibodies to citrullinated clusterin noted herein indicate this protein is playing an important role in early breaks in tolerance. In addition, histones in general (including H2, H4, and citrullinated forms) have been thought to play a role in the formation of neutrophil extracellular traps (NETs) in mucosal and systemic inflammation^42,43^; furthermore, NETosis and related histones may be an important part in triggering and/or propagating mucosal and systemic inflammation and potentially antigen generation in RA^44–46^. As such, future studies should evaluate the pathogenic significance of immunity to clusterin and histone H4 in pre-RA, especially as a potential factor in mucosal inflammation and pre-RA.

Of additional interest is the finding of significant elevations of T cells specific for citrullinated CILP antigens in the At-Risk participants compared to anti-CCP3(−) Controls while there were no significant differences between levels of antibodies to CILP between these groups; furthermore, in At-Risk participants compared to anti-CCP3(−) Controls there was no significant correlation between T cells specific for citrullinated CILP antigens and antibodies to CILP. In contrast, there were significant elevations of anti-citrullinated CILP antibodies in Early RA. While this may be the result of potential differences in the antigens detected in each of these experimental systems, this may also indicate that for some autoimmune responses to self-antigens, T cell reactivity precede the development of detectable autoantibodies, and potentially facilitate the later expansion of B cell production of autoantibodies.

In sum, this study identifies what are likely to be very early phenotypes associated with an anti-CCP3(+) “at-risk” state. Although prior analyses have reported mucosal alterations as well as global lymphocyte subset distribution differences in At-Risk individuals, ours is the first study to identify both significant pathway differences through epigenetic studies of cell subsets as well as autoantigen responses that may represent an “original sin”. In this scenario, cit-CILP and other reactivities (e.g., cit-clusterin, cit-histone H4) may represent early self-antigens to which tolerance is broken, after which epitope spreading occurs in both T and B cell responses. In this regard, while B and T cells exhibit exaggerated responses to only a subset of the known citrullinated autoantigens, they differ from one to another with respect to specificity. T cells respond to citrullinated CILP, whereas B cells respond to other citrullinated antigens (e.g., cit-clusterin, cit-histone H4). These reactivities may reflect inflammation and autoantigen exposure at their sites identified above, perhaps driven through mechanisms manifest by Th17.1 cells, or alternately may reflect the initial targets of molecular mimicry. Nevertheless, regardless of the origins of the responses, which are under investigation, they are currently intriguing self-antigen candidates for consideration in tolerance strategies. Finally, although by their nature cell subset-derived epigenetic changes do not address antigen specificity, the signaling pathways suggested are clearly focused on those of relevance to immune responses.

## Methods

### Study approval

The study was reviewed and approved by institutional review boards at all participating institutions including the University of Colorado, Denver, Colorado USA and Benaroya Research Institute, Seattle, Washington USA where participants were evaluated. In addition, the study protocols were approved at Stanford University, Palo Alto, California USA and the University of California, San Diego, San Diego, USA where only laboratory testing was performed. All participants completed a written informed consent process prior to study participation. All participants received compensation for their participation in study visits.

### Study participants

#### Identification of subjects

The Targeting Immune Responses for Prevention of Rheumatoid Arthritis (TIP-RA) cohort was designed to prospectively study individuals at high risk for developing RA (i.e., At-Risk) due to the presence at baseline of serum ACPA positivity in the absence of a history or physical examination evidence of IA at their baseline visit.

At-Risk individuals as well as anti-CCP3(−) control subjects were recruited through screening of health-fair participants, first-degree relatives of patients with RA, and individuals referred for evaluation to rheumatology clinics. In addition, individuals with Early RA were identified through rheumatology clinics. Of note, At-Risk and Early RA subjects were only included if they tested positive for anti-CCP3 on two occasions: at screening and at their baseline study visit; in addition, anti-CCP3(−) subjects were included if they tested negative for anti-CCP3 on two occasions: screening and baseline study visit. For individuals designated as Early RA, additional study requirements were that the baseline visit occur within 12 months of the first identification of inflammatory arthritis considered to be RA by a rheumatologist, and no disease-modifying anti-rheumatic drug (DMARD) therapy except a prednisone equivalent of less than 10 mg/day to optimize study of “early” clinically-apparent RA potentially prior to substantial modifications due to prolonged disease or treatments.

#### Clinical phenotyping

All subjects were evaluated at two study sites: the University of Colorado Anschutz Medical Campus, Aurora, Colorado, USA and the Benaroya Research Institute, Seattle, Washington USA, with recruitment from 2016-2018. All data included in these analyses were obtained at a baseline study visit for all participants. Clinical data was obtained using questionnaires that have been established in previous studies of individuals with classified RA as well as individuals who are at-risk for future RA^47^, and included self-report of sex assigned at birth. All subjects underwent a 66/68 joint examination for tender and swollen joints by a trained examiner. In addition, the absence of IA in the At-Risk and anti-CCP3(−) participants was confirmed on study-based physical examination as well as study questionnaires and review of their medical records; for the individuals with Early RA their diagnosis was confirmed using study based physical examination, as well as study questionnaires and review of their medical records.

#### Clinical autoantibody testing

The primary inclusion autoantibody biomarker was the anti-CCP3 assay (IgG, Inova Diagnostics Inc., San Diego, CA) with positivity determined by the manufacturer’s suggested cut-off level of >=20 units. Additional autoantibody testing including rheumatoid factor (RF) IgA and IgM (Inova Diagnostics Inc., San Diego, CA) with positivity determined using a local cut-off level that is equivalent to a level present in <2% of a non-RA population. All testing for these autoantibodies for all subjects was performed in the Exsera Biolabs at the University of Colorado Anschutz Medical Campus (www.exserabiolabs.org).

#### Final participants

We evaluated 97 participants who exhibited ACPA positivity by the commercial anti-CCP3 ELISA assay (IgG Inova Diagnostics, Inc., San Diego, CA; positive level >=20 units) who at their baseline study visit did not have historical or current examination evidence of inflammatory arthritis (IA) (66/68 joint count by a trained rheumatologist); these participants are designated herein as “At-Risk”. In addition, 172 anti-CCP3(−) individuals without IA or anti-CCP3 positivity were evaluated; these participants are designed anti-CCP3(−) controls. Finally, 62 individuals were enrolled who were anti-CCP3(+) with a clinical diagnosis of RA by a board-certified rheumatologist within 12 months of their initial study visit; these participants are designated “Early RA”. Of note, 61 (98.4%) of the Early RA participants met ACR/EULAR 2010 classification criteria at their baseline visit^3,48^, and their study visit was a mean of 55 days from the initial identification of IA by a rheumatologist.

The characteristics of these three groups at their baseline study visit are presented in Table 1. In addition, the subgroups of subjects who were evaluated in additional experiments (e.g., ACPA array, tetramer studies and epigenetic evaluations) are designated in Supplementary Fig. 2.

### Methylation

Genomic DNA was isolated from two independent cohorts of anti-CCP3(−) controls, At-Risk and Early RA individuals, and including only females to eliminate sex-related methylation differences (Supplementary Fig. 2). PBMC were separated by use of antibodies and magnetic beads into B, memory T and naïve T cell subsets for analyses. Cohort 1 was fully analyzed before the Cohort 2 was collected or DNA methylation assays performed. By having an initial dataset (cohort 1) and an independent confirmatory dataset (cohort 2), batch effects were minimized and the need for an interim analysis correction was eliminated.

*BeadChip analysis* DNA methylation level was measured using the Illumina Infinium MethylationEPIC Kit chip. The dataset was processed with the minfi package^49^ and normalized via the wateRmelon package in R^50^. Quality control steps based on probe detection *p*-value, methylation signal intensity, beta outliers, bisulfite conversion and bead count levels were performed using the minfi package and resulted in 1 sample and 4682 probes being removed from analysis. An additional filter to identify probes likely to overlap with a SNP was performed using the MethylToSNP package^51^; 480 probes were identified that potentially overlapped SNP. Of these, 325 probes were identified as having a high likelihood of overlapping SNP and were removed. Next, we removed any probe known to overlap a common SNP based on annotation in the IlluminaHumanMethylationEPICanno.ilm10b4.hg19 package using the dropLociWithSnps function in minfi^49^. Finally, we removed probes with ≥47 bp homology with an off-target site, previously annotated^52^. Of note, not all cell lineages and all participants had DNA methylation assays performed due to resource constraints, contributing to different numbers of samples in various lineages.

*DML, DMG and pathway identification* DML were identified using Welch’s *t*-test. Considering the limited number of DML identified original *p* values were not subjected to multiple testing corrections. To define a DML, *p* < 0.05 and differences of average β > 0.1 were used. DML were combined for both cohorts and mapped to gene promoter regions (−2500 to 500 bps from the transcription start site) and to gene bodies to define DMG. To identify differentially methylated pathways, DMG were mapped to biological pathways and pathways were selected based on DMG overrepresentation using the ReactomePA R package^53^.

*Dimension reduction analysis* Classical Multidimensional Scaling (MDS) algorithm was used for general non-linear dimension reduction on combined cohorts across all loci. Principal component analysis (PCA) was used to represent relationship among each blood cell type based on defined DML sets. M-values representing methylation intensities were used in the visualization of results.

*Hierarchical Clustering Analysis* All samples and DML for B cells, memory T cells and naïve T cells were clustered based on the union of the top 10% of DML, by importance, from random forest models separating anti-CCP3(−), At-Risk and Early RA samples using hierarchical clustering and plotted via the heatmap package with standard parameters in R. M-values representing methylation intensities were used in the visualization of results.

*Classification model* Pairwise one-vs-one random forest models were established to distinguish between anti-CCP3(−) control, At-Risk and Early RA samples in combined cohorts. Only DML previously selected for each comparison were used in the analysis. Samples for each model were separated into training (70%) and test (30%) data sets. Models were trained using 10-fold cross validation via the caret package in R^54^. For each model, a grid search was performed to tune hyperparameters and optimize accuracy. After training and validation, models achieved accuracy of 89.7%, 78.1%, and 96.8% when tasked with examining test data to distinguish between Early RA vs At-Risk, Early RA vs anti-CCP3(−) Controls and At-Risk vs anti-CCP3(−) Controls, respectively.

### Peptide-MHC Tmr generation

The citrullinated peptide sequence and location within each antigen are listed in Supplementary Table 2. Citrullinated peptides were selected based on established binding capacity to DR04:01 molecule and peripheral frequency as described previously^15,29^. Peptides were synthesized by Sigma and tetramers were generated by the BRI Tetramer Core as previously described^28,55^.

### Multi-color HLA class II tetramer staining

Multi-color HLA class II tetramer staining was performed as previously described^28,29^. In brief, a total of 30–40 million PBMC were thawed, rested at 37 °C for 2 h, and re-suspended in 200 µl of T cell media. Cells were then treated with Dasatanib for 10 min at 37 °C to prevent TCR internalization and incubated with 4.5 µl of each tetramer for 90 min at room temperature. Cells were then labeled with anti-PE, anti-APC, and anti-Myc magnetic beads (Miltenyi Biotec) for 20 min at 4 °C, enriched on a magnetic bead column according to the manufacturer’s instructions (Miltenyi Biotec). Before magnetic enrichment, 1% of the cell fraction was reserved as a pre-enrichment cell fraction to estimate total number of CD4 + T cells. Pre-enrichment cells and enriched cells were surfaced stained for 30 min at 4 °C with fluor-conjugated antibodies including CD4-V500, CD45RA-AF700, and CD38-BUV395 (all from BD Bioscience), and CD14-FITC, CD19-FITC, CXCR3-Pe/Cy7, CCR4-BV605, CCR6-BV785, and CCR7-PerCP/Cy5.5 (all from Biolegend) (Supplementary Table 3). Samples were collected on a BD FACS LSR Fortessa, and data were analyzed using FlowJo V10 (gating as shown in Supplementary Fig. 10) and Graphpad PRISM 7.0. The frequency of tetramer positive CD4 T cells is reported as “Frequency per Million CD4 T cells” and calculated as follows: F = (1,000,000 X tetramer positive events from enriched sample)/(100 X number of CD4+ cells from the non-enriched fraction).

### DISCOV-R computational analyses

The DISCOV-R computational analyses were performed as recently described^31^. In brief, FCS files prepared above were analyzed using custom R scripts (https://github.com/BenaroyaResearch/Buckner_Linsley_Cit-specific) based on the flowCore^56^, Rtsne^57,58^, and cytofkit packages^59^. Tmr+ events were concatenated to total CD4 + T cell FCS files for each sample prior to arcsinh transformation using parameters: a = 0, b = 1/150. Subsequently, t-SNE analysis and Rphenograph clustering (Individual Clusters) were performed for each sample using the following 6 phenotypic markers: CD45RA, CD38, CCR4, CCR6, CXCR3, and CCR7. Cluster alignment of Individual Clusters with >1% frequency was performed across individuals by hierarchically metaclustering the z-score values of each marker’s expression compared to each subject’s total CD4 + T cells, with Euclidean distance and Ward’s method used to assess phenotypic similarity; heatmaps were generated in R using the ComplexHeatmap package^60^. The dendrogram was cut into segments using cutree from the stats R package^61^ and the resulting cluster assignments (Aligned Clusters) were applied to the individual samples. Total CD4+ and Tmr+ T cells were counted for each aligned cluster in each subject, and their frequency was calculated.

### Antibody-antigen array testing and analyses

Serum levels of antibodies targeting 41 candidate RA-associated autoantigens were measured using a custom bead-based immunoassay on the Luminex platform as previously described^20^. Of the 41 antigens in the bead-based array, 27 are citrullinated and 14 are native. Ten CILP peptides were included on the arrays, and were selected based on the citrullinated versions exhibiting reactivity to antibodies in established RA. The list of autoantigens is provided in Supplementary Data 3, and peptides were synthesized with terminal biotins and couples using bead regions pre-conjugated with streptavidin (Bio-Rad Laboratories). An anti-IgG secondary antibody was used to detect autoantibody binding. Results of arrays are reported as mean fluorescence units.

We performed analyses comparing antibodies as levels an as positive/negative between groups, with positivity of an antibody determined as a level >=3 standard deviations above the mean level of all of the anti-CCP3(−) Control samples. In analyses of the antibodies as levels, we evaluated levels of antibodies to individual antigens; in addition, to optimize signal as many peptides are from the same protein, we aggregated similar peptides and citrullinated status together by summing the antibody levels (e.g., summed levels of antibodies to cit-fibrinogen containing antigens) as well as counting the number of positive antibodies.

For antibody levels, a multivariable linear regression was performed, while a logistic regression or Poisson regression was performed for the positivity analyses (respective for either a singular antibody positivity or a summed count of positivity). In all analyses (i.e., antibodies as levels, or as positive/negative), we modeled group status (anti-CCP3(−) Controls, At-Risk or Early RA) predicting ACPA levels/positivity while adjusting for age, sex, and smoking status (ever/never), and pairwise comparisons of At-Risk to anti-CCP3(−) subjects and At-Risk to Early RA subjects are reported. Furthermore, we report results using a *p* < 0.05 as significant; in addition, to address multiple comparisons, following ref. ^62^, we also report results as significant using a false-discovery rate (FDR)-adjusted *p*-value of 0.05. Finally, correlations between number of T cells reactive to CILP and antibodies to CILP were performed using Spearman technique.

We also performed additional analyses stratifying subjects by the presence/absence of the *0401 haplotype; in these stratified analyses, we also performed the same regression models as mentioned above, adjusted by age, sex and smoking status.

### Reporting summary

Further information on research design is available in the Nature Portfolio Reporting Summary linked to this article.

### Supplementary information

Supplementary Information

Description of Additional Supplementary Files

Supplementary Data 1

Supplementary Data 2

Supplementary Data 3

Supplementary Data 4

Supplementary Data 5

Supplementary Data 6

Reporting Summary

### Source data

Source Data

## Data Availability

Methylation: The methylation data generated in this study (including for Fig. 1, Supplementary Fig. 1 and Supplementary Table 1 and Supplementary Data 1 and 2) have been deposited in NCBI’s Gene Expression Omnibus and are accessible via the accession number GSE230446. T cell assays: The T cell data generated in this study (including Figs. 2 and 3, and Supplementary Fig. 3−5) have been deposited in github https://github.com/BenaroyaResearch/TIP_RA_cross_sectional/blob/main/James_et_al_TIP_RA_cross_sectional_source_data.xlsx. Autoantibody array: The raw data used for the autoantibody array analyses (including for Supplementary Figs. 6–9 and Supplementary Data 3–6) are available at: https://github.com/Vanderll/TIPRA_baseline. (version v1.1) 10.5281/zenodo.8433369. Clinical data: A portion of clinical data (including for Table 1) is available at https://github.com/Vanderll/TIPRA_baseline. (version v1.1) 10.5281/zenodo.8433369. However, additional clinical data generated in this study are protected and are not available due to data privacy laws. Source data are provided with this paper.

## Code availability

Methylation: The code to reproduce our methylation data analysis and arrange the related figures in this study (including for Fig. 1, Supplementary Fig. 1 and Supplementary Table 1 and Supplementary Data 1 and 2) can be found at https://github.com/Wang-lab-UCSD/TIP-RA-CS. Given the set of scripts and environments provided, only minimal changes (such as file paths) are necessary to reproduce the analyses of this work. T cell assays: The code for the T cell analyses (including Figs. 2 and 3 and Supplementary Figs. 3−5) is available at: https://github.com/BenaroyaResearch/TIP_RA_cross_sectional. Autoantibody array: The code for the autoantibody array analyses (including for Supplementary Figs. 6–9 and Supplementary Data 3–6) are available at: https://github.com/Vanderll/TIPRA_baseline. (version v1.1) 10.5281/zenodo.8433369.
