# Supplementary material for: Multifaceted immune dysregulation characterizes individuals at-risk for rheumatoid arthritis
Source: Nat Commun. 2023 Nov 22;14:7637. doi: 10.1038/s41467-023-43091-8 (PMC10665556; doi:10.1038/s41467-023-43091-8)
Supplement: Supplementary file 1 — Supplementary Information [file 41467_2023_43091_MOESM1_ESM.pdf]

SUPPLEMENTAL FIGURES AND TABLES

Multifaceted Immune Dysregulation Characterizes Individuals At-Risk for Rheumatoid Arthritis

Eddie A. James[1, †], V. Michael Holers[2, † ∞], Radhika Iyer[3†,], E. Barton Prideaux[4†,], Navin L. Rao[5], Cliff Rims[1], Virginia S. Muir[1], Sylvia E. Posso[1], Michelle S. Bloom[3], Amin Zia[3], Serra E. Elliott[3], Julia Z. Adamska[3], Rizi Ai[4], Camille Brewer[3], Jennifer A. Seifert[2], LauraKay Moss[2], Saman Barzideh[2], M. Kristen Demoruelle[2], Christopher C. Striebich[2], Yuko Okamoto[2,6], Enkhtsogt Sainbayar[2], Alexandra A. Crook[2], Ryan A. Peterson[7], Lauren A. Vanderlinden[7], Wei Wang[4,8], David L. Boyle[8], William H. Robinson[3, †], Jane H. Buckner[1, †], Gary S. Firestein[8, †], Kevin D. Deane[2, †]

Affiliations

1. Benaroya Research Institute, Seattle, WA 98101, USA
2. Division of Rheumatology, University of Colorado Anschutz Medical Campus, Aurora, CO 80045, USA
3. Division of Immunology and Rheumatology, Stanford University, Stanford, CA 94304, USA and VA Palo Alto Health Care System, Palo Alto, CA 94550, USA
4. Department of Chemistry and Biochemistry, University of California, San Diego, La Jolla, CA, 92093, USA
5. Janssen Research and Development, Spring House, PA 19477, USA
6. Division of Rheumatology, Department of Internal Medicine, Tokyo Women's Medical University School of Medicine, Tokyo, Japan
7. Department of Biostatistics and Informatics, Colorado School of Public Health, University of Colorado Anschutz Medical Campus, Aurora, CO 80045, USA

8. Department of Cellular and Molecular Medicine, University of California, San Diego, La Jolla, CA, 92093, USA
9. Division of Rheumatology, Allergy and Immunology, University of California, San Diego, La Jolla, CA 92093, USA

†Equally-contributing authors

∞Corresponding author:
V. Michael Holers
Division of Rheumatology, Box B115
1775 Aurora Ct.
Aurora, CO 80045, USA
michael.holers@cuanschutz.edu; 303-724-7605 (tel)

Supplemental Figures

Supplemental Figure 1. Heatmap with hierarchical clustering illustrating the methylation intensities of DMLs from the union of top 10% most important features selected by random forest models separating anti-CCP3(-), At-Risk and Early RA samples.

Supplemental Figure 2. Consort diagram of participant testing for each type of analysis (e.g. ACPA array, tetramer analyses/T cell phenotyping and DNA methylation).

Supplemental Figure 3. Determining the total CD4+ T cell landscape.

Supplemental Figure 4. Observed phenotype of Influenza specific CD4+ T cells.

Supplemental Figure 5. Observed phenotype of Total CD4+ T cells.

Supplemental Figure 6. Heatmap demonstrating positive/negative results for each antibody by participant group and including all participants.

Supplemental Figure 7. Heatmap of anti-CILP antibody levels across groups and in relationship to CILP-reactive T cells identified through tetramer analyses.

Supplemental Figure 8. Heatmap demonstrating positive/negative results for each antibody by participant groups among those who were positive for at least one HLA DR4*0401 allele.

Supplemental Figure 9. Heatmap demonstrating positive/negative results for each antibody by participant groups among those who were negative for any allele containing HLA DR4*0401.

Supplemental Figure 10. Gating strategy to identify T cell frequencies and phenotypes.

Supplemental Tables

Supplemental Table 1. Differentially methylated pathways comparing anti-CCP3(-), At-Risk and Early RA in B cells and naïve T cells.

Supplemental Table 2. Peptides and staining label for Tmr studies.

Supplemental Table 3. Antibodies, staining label and commercial source for phenotyping studies.

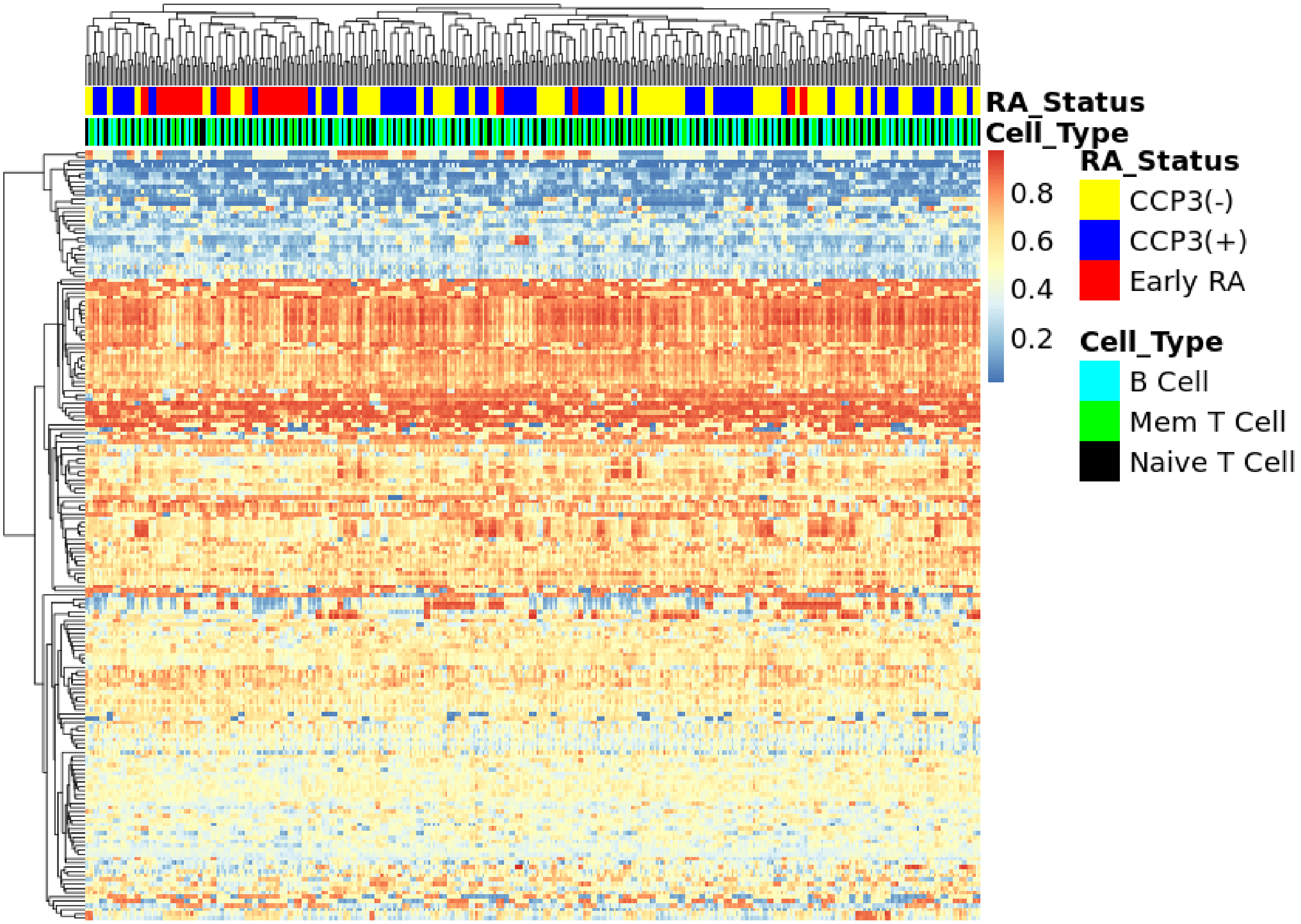

Supplemental Figure 1. Heatmap with hierarchical clustering illustrating the methylation intensities of DMLs from the union of top 10% most important features selected by random forest models separating anti-CCP3(-), At-Risk and Early RA samples. Hierarchical clustering clusters samples by clinical status, especially Early RA, with reasonable accuracy though visual differences between groups are subtle. Source data are provided as a Source Data file.Abbreviations: Anti-CCP3=anti-cyclic citrullinated peptide antibody, RA=rheumatoid arthritis

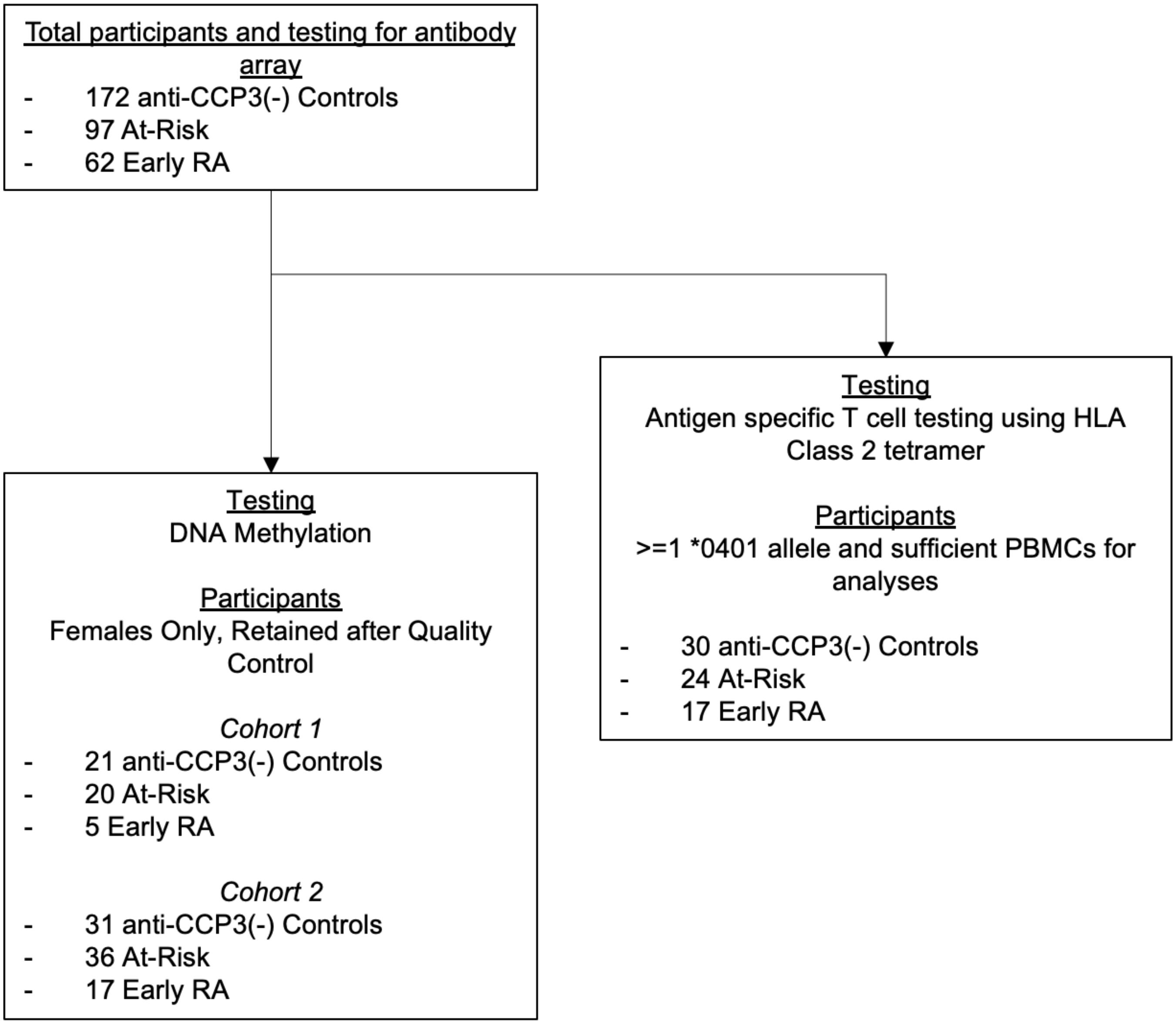

Supplemental Figure 2. Consort diagram of participant testing for each type of analysis (e.g. antibody-antigen array, tetramer analyses/T cell phenotyping and DNA methylation). Abbreviations: ACPA=anti-bodies to citrullinated protein antigens; RA=rheumatoid arthritis; HLA=human leukocyte antigen; DNA=deoxyri-bonucleic acid

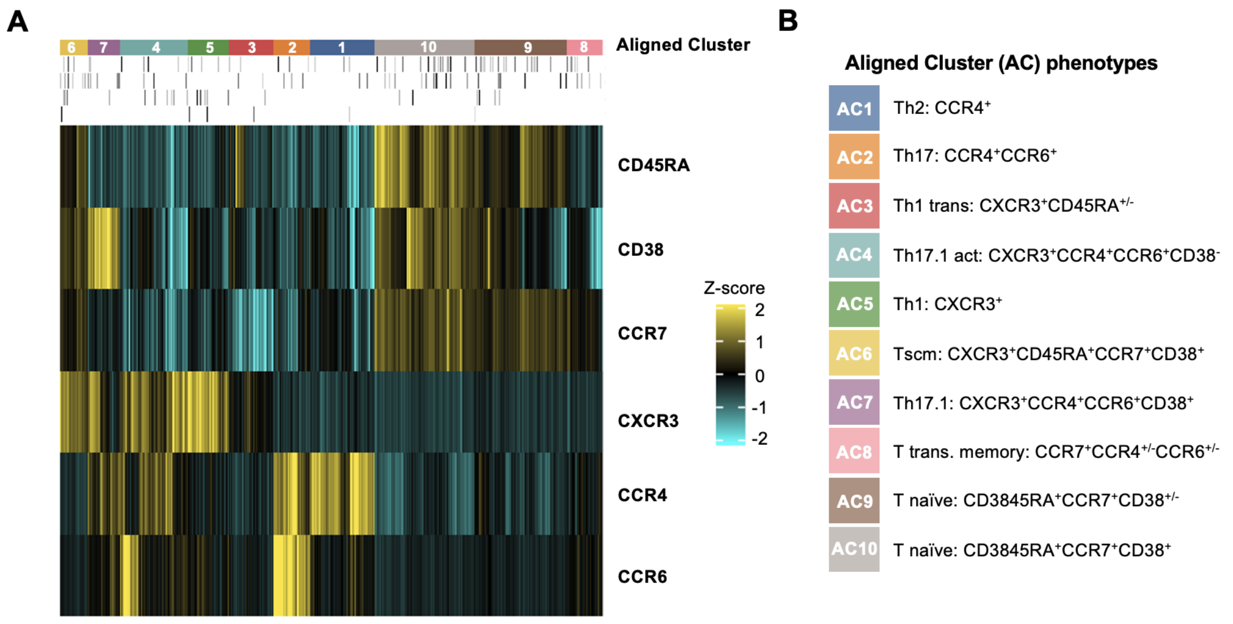

Supplemental Figure 3. Determining the Total CD4+ T cell Landscape. (A) Heat map showing all CD4+ T cell clusters from 30 Anti-CCP3(-) Controls, 24 At-Risk, and 17 Early RA participants, hierarchically clustered (Euclidean distance, Ward's minimum variance linkage) by expression of six phenotyping markers as a z-score comparing mean cluster intensity to total CD4 T cell intensity for each subject. The resulting dendrogram is divided into ten aligned clusters with color bar across top indicating aligned cluster assignment. (B) Suggested lineages of aligned clusters (AC) representing distinct surface phenotype groups within the CD4+ T cell landscape. Source data are provided as a Source Data file.

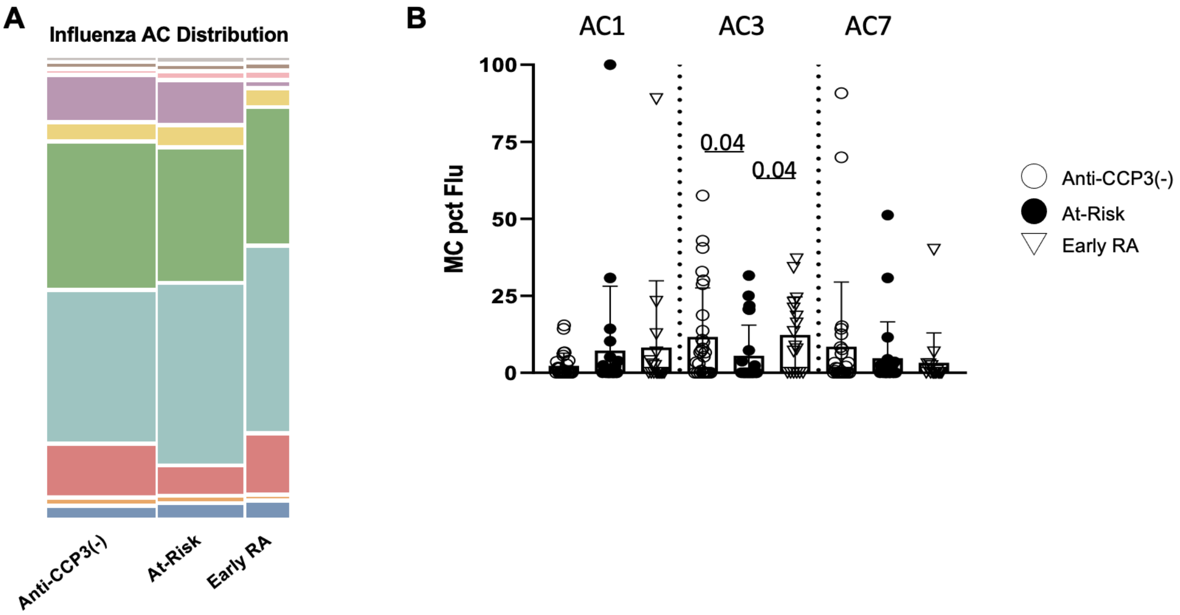

Supplemental Figure 4. Observed phenotype of Influenza specific CD4+ T cells. (A) Stacked box plots show the T cell surface phenotype aligned cluster (AC) distribution of influenza specific T cells. (B) A lower proportion of influenza reactive T cells resided within AC3 (a transitional Th1 cluster) in At-Risk participants as compared with Early RA participants and Anti-CCP3(-) controls (p=0.044). Subject counts were anti-CCP3(-) N=28, at-risk N=24, and early-RA N=17. Error bars indicate standard deviation. P-values indicate unpaired comparisons using Wilcoxon's nonparametric two-tailed test. Source data are provided as a Source Data file. AC surface phenotype group definitions: AC1= Th2; AC3=Th1 transitional; AC7=Th17.1 (for additional detail on AC definitions, see Supplemental Figure 3).

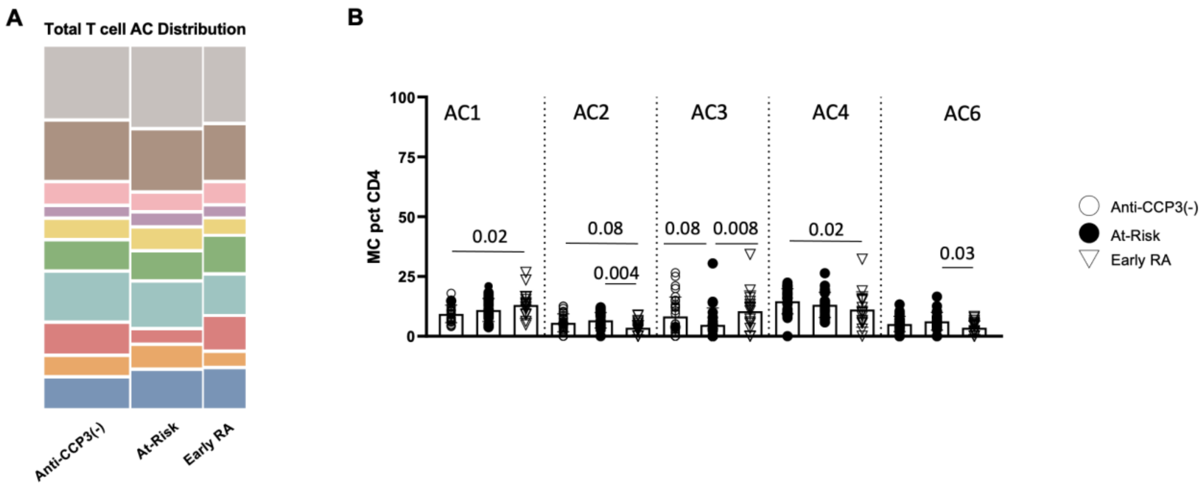

Supplemental Figure 5. Observed phenotype of Total CD4+ T cells. (A) Stacked box plots show the T cell surface phenotype aligned cluster (AC) group distribution of total CD4+ T cells and from Anti-CCP3(-) Controls, At-Risk, and Early RA participants. (B) Several modest differences were observed in the AC distribution of total CD4+ T cells; these include a higher proportion within AC1 for Early RA participants; a lower proportion within AC2, AC4, and AC6 for Early RA participants, and a lower proportion within AC3 for At-Risk participants. Subject counts were anti-CCP3(-) N=29, at-risk N=24, and early-RA N=17. Error bars indicate standard deviation. P-values indicate unpaired comparisons using Wilcoxon's nonparametric two-tailed test. Source data are provided as a Source Data file. AC surface phenotype group definitions: AC1= Th2; AC2=Th17; AC3=Th1 transitional; AC4=Th17.1 activated; AC6=Tscm (for additional details on AC definitions, see Supplemental Figure 3).

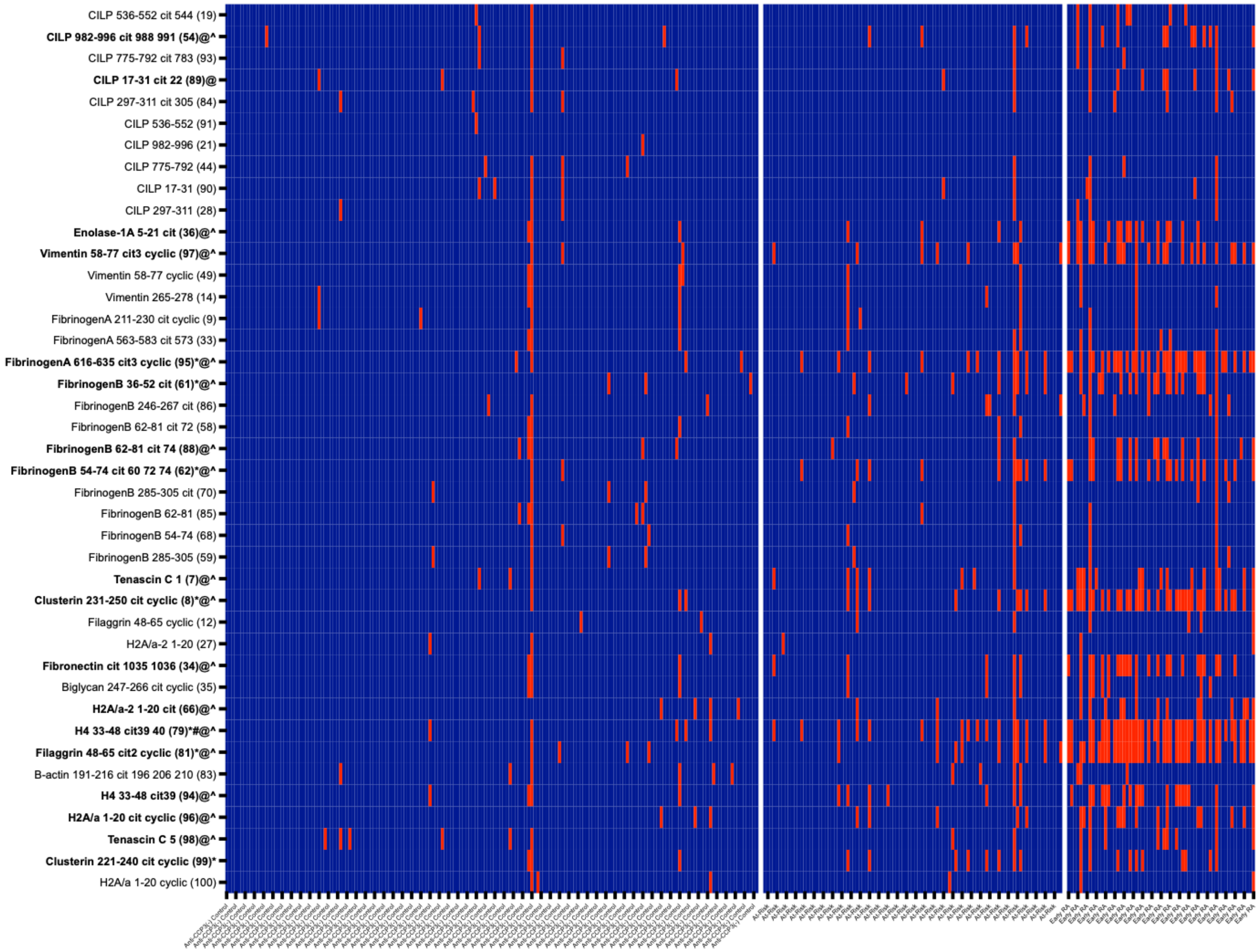

Supplemental Figure 6. Heatmap demonstrating positive/negative results for each antibody by participant group and including all participants [anti-CCP3(-) Controls n=172; At-Risk n=97; Early RA n=62]. Each antibody (rows) is given positive/negative status based on a level >=3 standard deviations above the levels in the anti-CCP3(-) Controls. Each column represents a single participant (every 3rd participant labelled). Pairwise comparisons were made between anti-CCP(-) Controls and At-Risk participants, and At-Risk and Early RA participants using regression analyses and adjusting for age, sex/gender, and current smoking. To determine significant differences, p-values of <0.05 were used, as well as a stricter false discovery rate (FDR) adjusted p-value to adjust for multiple comparisons. The antibodies that were significantly different in positivity rates between groups are bolded. In particular, in comparisons between At-Risk participants and anti-CCP(-) Controls, antibodies marked with (*) were positive in a significantly higher number of At-Risk participants at p<0.05, and those marked with (#) were also significant at the FDR-adjusted p-value. In comparisons between Early RA and At-Risk participants, antibodies marked with (@) were positive in a significantly higher number of Early RA participants at p<0.05, and those marked with (^) were also significant at the FDR-adjusted p-value. Additional findings from these analyses are presented in Supplemental Table 6. Source data are provided as a Source Data file. Abbreviations: Cartilage intermediate layer protein (CILP); Fibrinogen alpha chain (fibrinogenA); Fibrinogen beta chain (fibrinogenB); Histone H4 (H4); Histone 2A (H2A); beta actin (B-actin).

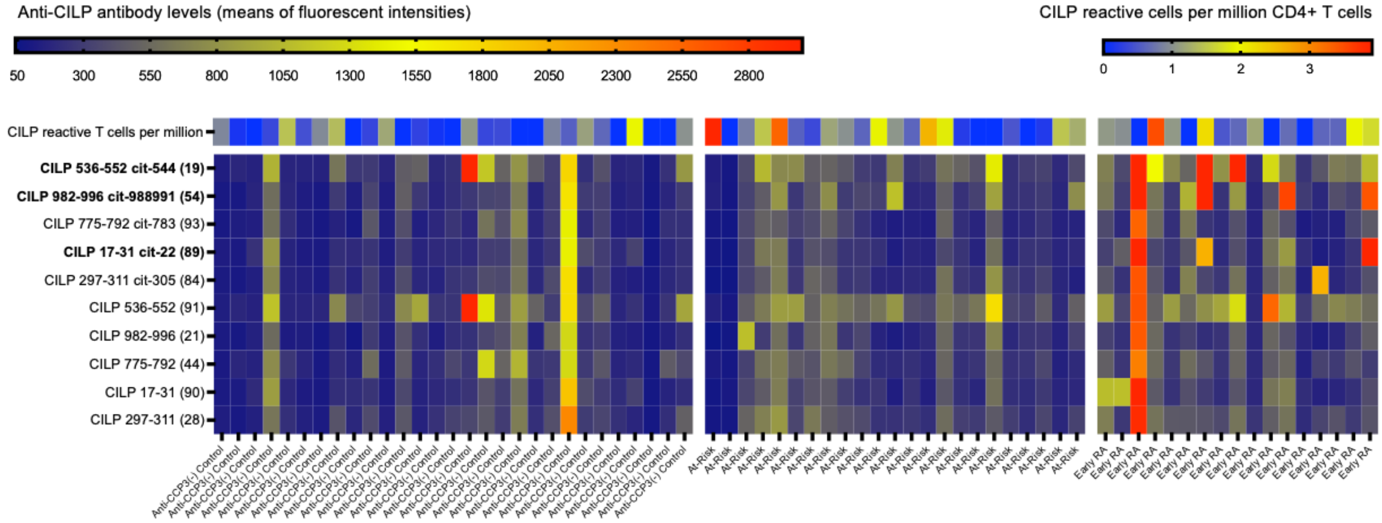

Supplemental Figure 7. Heatmap of anti-CILP antibody levels across groups and in relationship to CILP-reactive T cells identified through tetramer analyses. The columns represent participants within each group who had the presence of at least one HLA DR4*0401 allele and also underwent testing using tetramer analyses for reactivity to citrullinated and non-citrullinated cartilage intermediate layer protein (CILP) (anti-CCP3(-) Controls n=30, At-Risk n=24 and Early RA n=17). In separate analyses (see Supplemental Table 6), mean antibody levels to the three citrullinated versions of CILP (bolded) were elevated in Early RA compared to At-Risk participants; however, there were no significant differences in anti-CILP levels between At-Risk and Anti-CCP3(-) Controls. In addition, there were no significant correlations between levels of CILP-reactive T cells and antibody levels (Spearman correlation testing, data not shown). Source data are provided as a Source Data file.Abbreviations: Cit=citrullinated; Anti-CCP3=anti-cyclic citrullinated peptide antibody; RA=rheumatoid arthritis

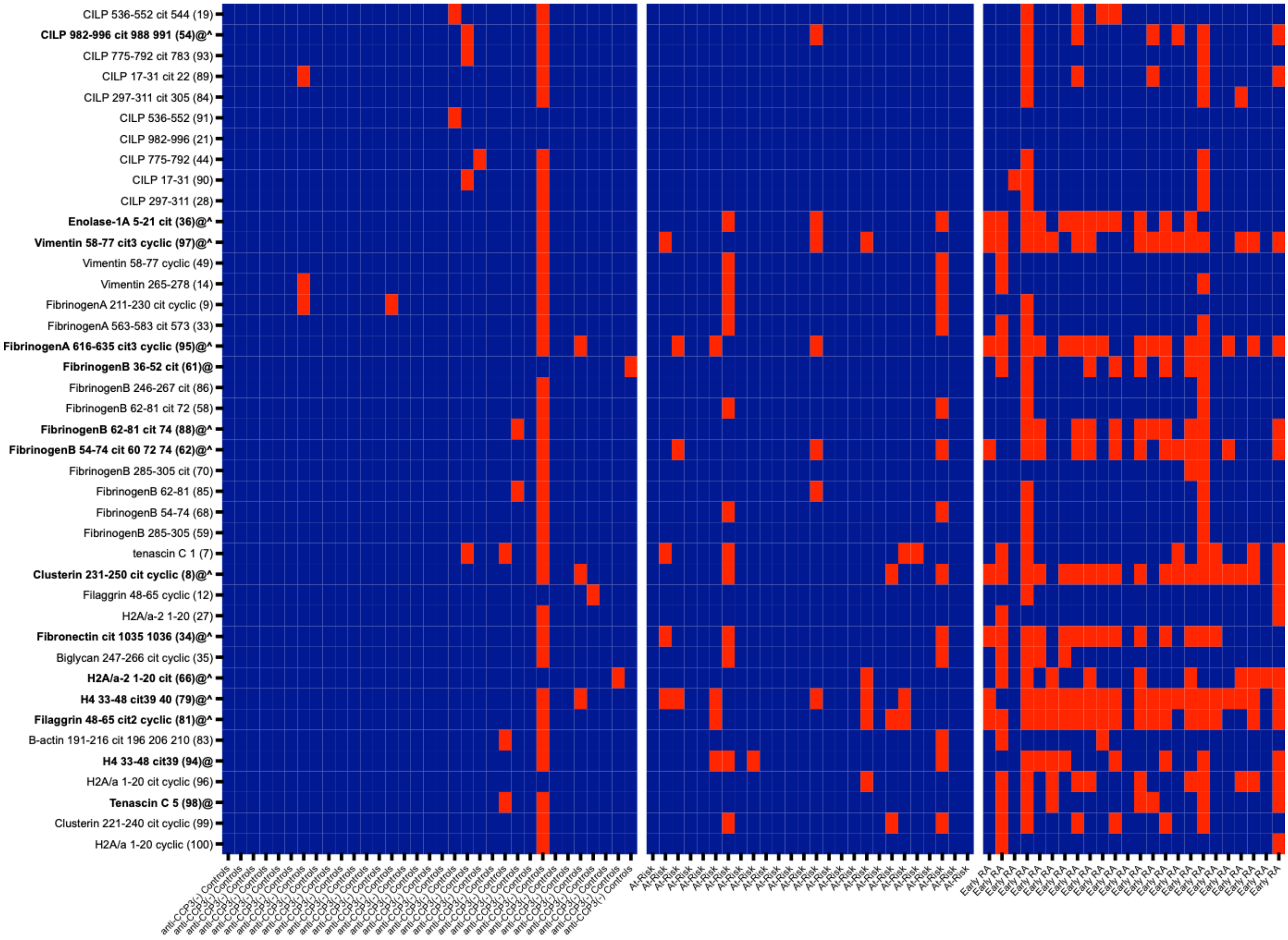

Supplemental Figure 8. Heatmap demonstrating positive/negative results for each antibody by participant groups among those who were positive for at least one HLA DR4*0401 allele [anti-CCP3(-) Controls n=33; At-Risk n=26; Early RA n=24]. Each antibody (rows) is given positive/negative status based on a level >=3 standard deviations above the levels in the anti-CCP3(-) Controls. Each column represents a single participant. Pairwise comparisons were made between anti-CCP(-) Controls and At-Risk participants, and At-Risk and Early RA participants using regression analyses and adjusting for age, sex/gender, and current smoking. To determine significant differences, p-values of <0.05 were used, as well as a stricter false discovery rate (FDR) adjusted p-value to adjust for multiple comparisons. The antibodies that were significantly different in positivity rates between groups are bolded. In comparisons between At-Risk participants and anti-CCP(-) Controls, no antibodies had significantly different positivity rates at either p<0.05 or FDR-adjusted p-value levels. In comparisons between Early RA and At-Risk participants, antibodies marked with (@) were positive in a significantly higher number of Early RA participants at p<0.05, and those marked with (^) were also significant at the FDR-adjusted p-value. Additional findings from these analyses are presented in Supplemental Table 8. Source data are provided as a Source Data file. Abbreviations: HLA=Human Leukocyte Antigen; cartilage intermediate layer protein (CILP); Fibrinogen alpha chain (fibrinogenA); Fibrinogen beta chain (fibrinogenB); Histone H4 (H4); Histone 2A (H2A); beta actin (B-actin).

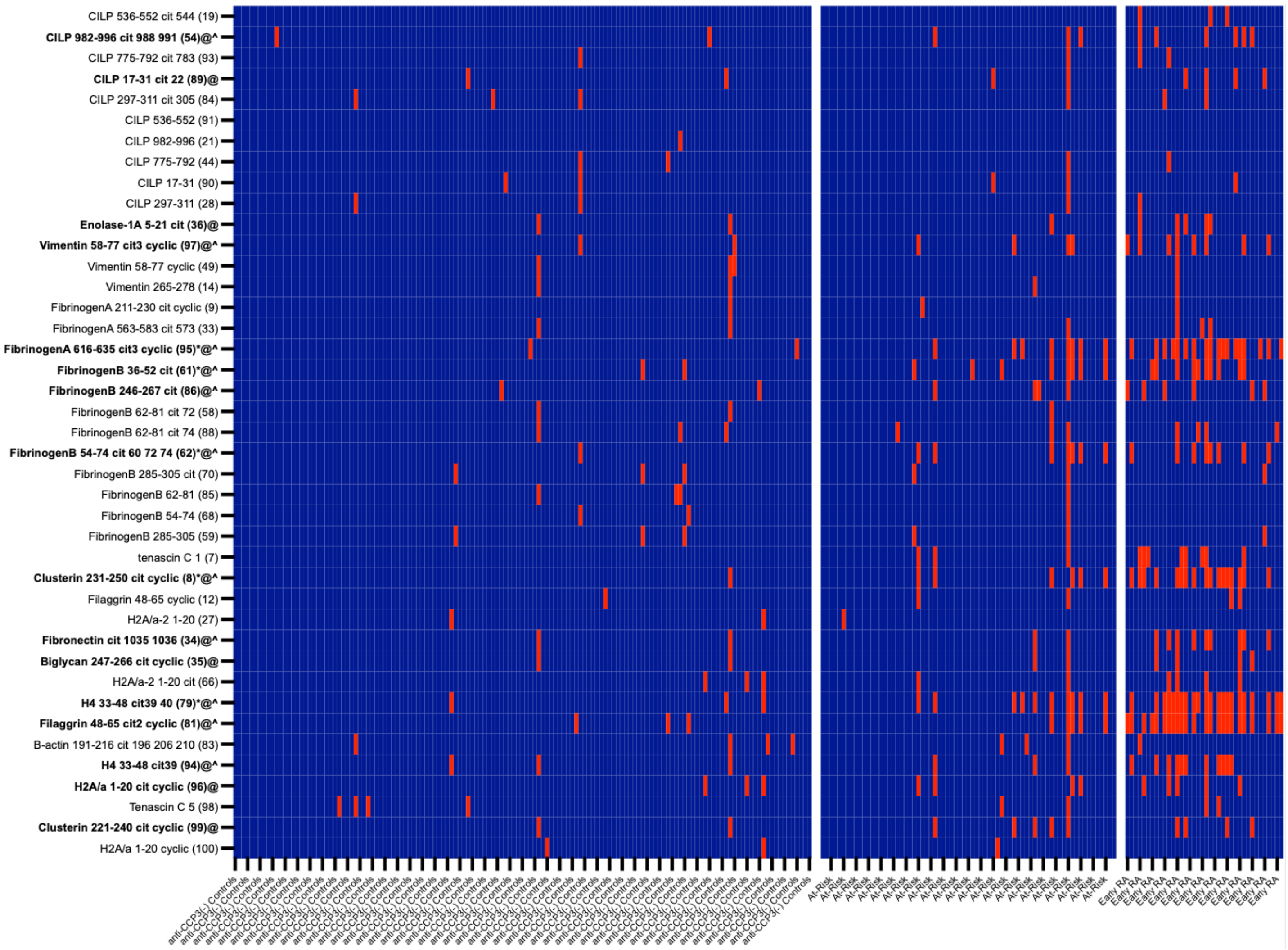

Supplemental Figure 9. Heatmap demonstrating positive/negative results for each antibody by participant groups among those who were negative for any allele containing HLA DR4*0401 [anti-CCP3(-) Controls n=139; At-Risk n=71; Early RA n=38]. Each antibody (rows) is given positive/negative status based on a level >=3 standard deviations above the levels in the anti-CCP3(-) Controls. Each column represents a single participant (every 3rd participant is labelled). Pairwise comparisons were made between anti-CCP(-) Controls and At-Risk participants, and At-Risk and Early RA participants using regression analyses and adjusting for age, sex/gender, and current smoking. To determine significant differences, p-values of <0.05 were used, as well as a stricter false discovery rate (FDR) adjusted p-value to adjust for multiple comparisons. The antibodies that were significantly different in positivity rates between groups are bolded. In comparisons between At-Risk participants and anti-CCP(-) Controls, antibodies marked with (*) were positive in a significantly higher number of At-Risk participants at p<0.05, and those marked with (#) were also significant at the FDR-adjusted p-value. In comparisons between Early RA and At-Risk participants, antibodies marked with (@) were positive in a significantly higher number of Early RA participants at p<0.05, and those marked with (^) were also significant at the FDR-adjusted p-value. Additional findings from these analyses are presented in Supplemental Table 8. Source data are provided as a Source Data file. Abbreviations: HLA=Human Leukocyte Antigen; cartilage intermediate layer protein (CILP); Fibrinogen alpha chain (fibrinogenA); Fibrinogen beta chain (fibrinogenB); Histone H4 (H4); Histone 2A (H2A); beta actin (B-actin).

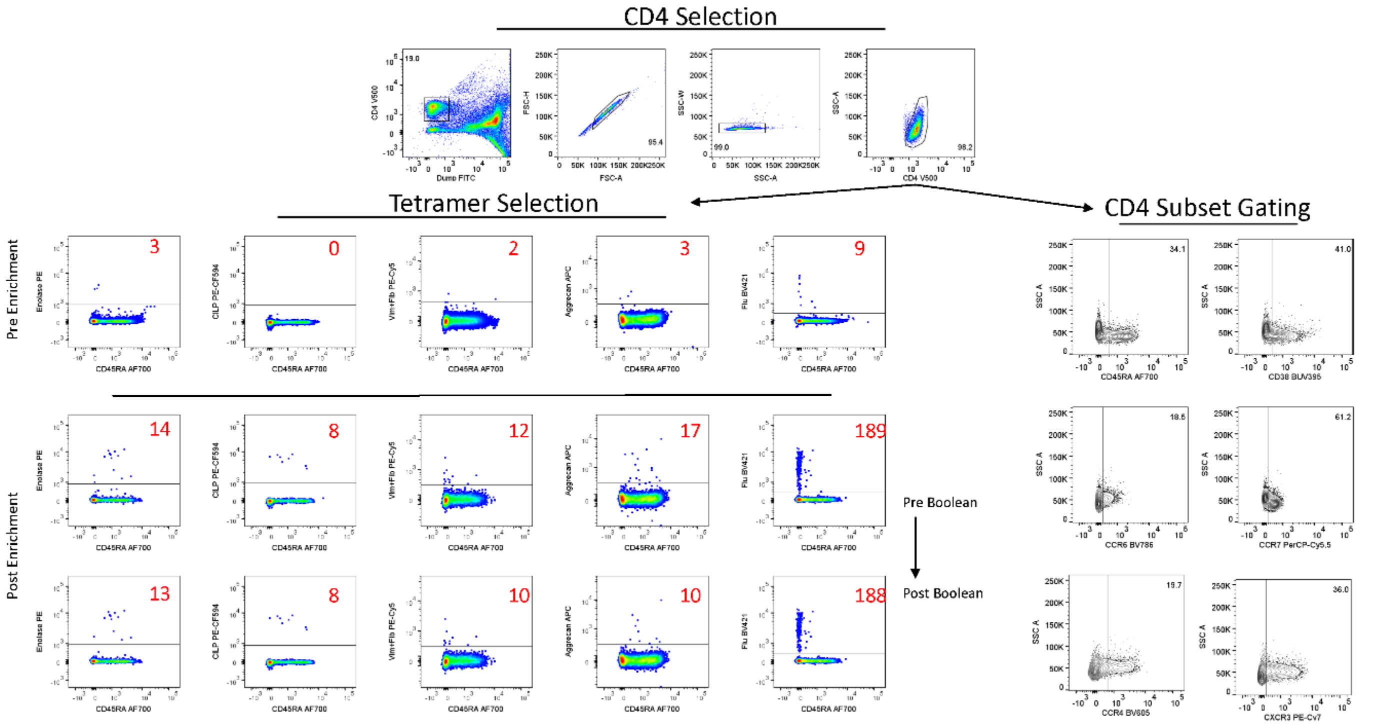

Supplemental Figure 10. Gating strategy to identify T cell frequencies and phenotypes. <u>CD4 Selection:</u> For tetramer assays, live CD4+ T cells were selected by first gating on the CD4+ Dump negative population (this channel contains anti-CD14, anti-CD19 and a dead cell exclusion dye). Two gates (FSC-H versus FSC-A and SSC-W versus SSC-A) were then applied to confidently gate singlets followed by a CD4 versus SSC-A "clean-up" gate. <u>Tetramer Selection:</u> Tetramer positive events were defined by passing all CD4+ T cells to a CD45RA versus tetramer gate for each tetramer channel (PE, PE-CF594, PE-Cy5, APC, and BV421). A threshold gate was then placed above the main population for each pre-enrichment sample. For each post-enrichment sample, events above the threshold gate were Boolean gated to eliminate events with positive signal on more than one tetramer channel. The post Boolean events were defined as the tetramer positive population and used to calculate frequencies. In the example shown, red numbers indicate the tetramer positive event count before and after Boolean gating. <u>CD4 Subset Gating:</u> Thresholds for cell phenotyping were defined by passing all CD4+ T cells from the pre-enrichment sample to an SSC-A versus cell surface marker plot for each marker (CD45RA, CD38, CXCR3, CCR4, CCR6, and CCR7) and bisecting the negative and positive populations using a contour plot. These thresholds were applied to each tetramer positive population to assess expression (or lack of expression) for each marker of interest. As this is a gating strategy, source data is from flowjo workspace.

Supplemental Table 1. Significantly (false discovery rate [FDR] < 0.10) differentially methylated pathways comparing anti-CCP3(-), At-Risk and Early RA in B cells, memory T cells, and naïve T cells. Source data are provided as a Source Data file

B Cells

Pathway Name	P-Value	FDR	DMGs
Collagen chain trimerization	7.26E-05	3.35E-02	COL13A1, COL22A1, COL4A2, COL5A3, 1ABR8
Collagen formation	2.75E-04	4.96E-02	COL13A1, COL22A1, COL4A2, COL5A3, 1ABR8, CTSB
Collagen degradation	4.35E-04	4.96E-02	COL13A1, COL4A2, COL5A3, 1ABR8, CTSB
cGMP effects	4.96E-04	4.96E-02	KCNMA1, PDE9A, PRKG1
Collagen biosynthesis and modifying enzymes	5.38E-04	4.96E-02	COL13A1, COL22A1, COL4A2, COL5A3, 1ABR8
NCAM1 interactions	7.91E-04	6.08E-02	CACNA1D, COL4A2, COL5A3, 1ABR8
Nitric oxide stimulates guanylate cyclase	1.30E-03	8.59E-02	KCNMA1, PDE9A, PRKG1

Memory T Cells

Pathway Name	P-Value	FDR	DMGs
Translocation of ZAP-70 to Immunological synapse	1.32E-10	1.03E-07	CD247, CD3D, CD3E, CD3G, HLA-DQB1, HLA-DQB2, HLA-DRB1, LCK
Phosphorylation of CD3 and TCR zeta chains	5.37E-10	2.10E-07	CD247, CD3D, CD3E, CD3G, HLA-DQB1, HLA-DQB2, HLA-DRB1, LCK
PD-1 signaling	8.13E-10	2.12E-07	CD247, CD3D, CD3E, CD3G, HLA-DQB1, HLA-DQB2, HLA-DRB1, LCK
Generation of second messenger molecules	2.62E-08	5.13E-06	CD247, CD3D, CD3E, CD3G, HLA-DQB1, HLA-DQB2, HLA-DRB1, LCK
Costimulation by the CD28 family	7.49E-07	1.17E-04	CD247, CD3D, CD3E, CD3G, HLA-DQB1, HLA-DQB2, HLA-DRB1, LCK, RAC1
Downstream TCR signaling	1.44E-05	1.88E-03	CD247, CD3D, CD3E, CD3G, HLA-DQB1, HLA-DQB2, HLA-DRB1, LCK, TRAT1
TCR signaling	7.25E-05	8.12E-03	CD247, CD3D, CD3E, CD3G, HLA-DQB1, HLA-DQB2, HLA-DRB1, LCK, TRAT1
RUNX1 and FOXP3 control the development of regulatory T lymphocytes (Tregs)	3.58E-04	3.51E-02	CR1, IFNG, IL2
Interferon gamma signaling	4.28E-04	3.73E-02	HLA-DQB1, HLA-DQB2, HLA-DRB1, HLA-E, IFNG, JAK1, SOCS1
Interleukin-2 signaling	6.43E-04	5.04E-02	IL2, JAK1, LCK
Signaling by NOTCH1	7.91E-04	5.21E-02	DLK1, DTX1, HDAC4, HDAC5, ITCH, NOTCH1
Interferon Signaling	7.97E-04	5.21E-02	HLA-DQB1, HLA-DQB2, HLA-DRB1, HLA-E, IFITM1, IFITM2, IFNG, JAK1, RAE1, SOCS1
Regulation of IFNG signaling	1.04E-03	6.06E-02	IFNG, JAK1, SOCS1
Activated NOTCH1 Transmits Signal to the Nucleus	1.08E-03	6.06E-02	DLK1, DTX1, ITCH, NOTCH1
Interleukin-12 family signaling	1.53E-03	8.00E-02	IFNG, IL23R, JAK1, LMNB1, MTAP
ROS and RNS production in phagocytes	1.91E-03	9.37E-02	ATP6V1A, MPO, SLC11A1, TCIRG1

Naïve T Cells

Pathway Name	P-Value	FDR	DMGs
Translocation of ZAP-70 to Immunological synapse	4.05E-04	9.17E-02	HLA-DQB1, HLA-DQB2, HLA-DRB1
Phosphorylation of CD3 and TCR zeta chains	6.33E-04	9.17E-02	HLA-DQB1, HLA-DQB2, HLA-DRB1
PD-1 signaling	7.24E-04	9.17E-02	HLA-DQB1, HLA-DQB2, HLA-DRB1

**Supplemental Table 2.** Peptides and staining label for Tmr studies.		
**Epitope**	**Sequence**	**Label**
**Enolase-11**	IFDS[Cit]GNPTVEVDLF	PE
**Enolase-26**	TSKGLF[Cit]AAVPSGAS	PE
**Enolase-326**	K[Cit]IAKAVNEKSCNCL	PE
**CILP-297**	ATIKAEFV[Cit]AETPYM	PE-CF594
**CILP-982**	GKLYGI[Cit]DV[Cit]STRDR	PE-CF594
**α Fibrinogen-24**	EGDFLAEGGGV[Cit]GPR	PE-Cy5
**β Fibrinogen-69**	GY[Cit]A[Cit]PAKAAAT	PE-Cy5
**Vimentin-59**	GVYAT[Cit]SSAV[Cit]L[Cit]SSVPGVR	PE-Cy5
**Vimentin-418**	SSLNL[Cit]ETNLDSL	PE-Cy5
**Aggrecan-225**	DEFPGV[Cit]TYGI[Cit]DTNETYDV	APC
**Aggrecan-553**	PGV[Cit]TYGV[Cit]PSTETYDVY	APC
**Influenza MP-97**	VKLYRKLKREITFHGAKEIS	BV421
**Abbreviations:** Tmr=tetramer; CILP=cartilage intermediate layer protein; [Cit]=Citrulline; PE=Phycoerythrin; PE-CF594=Phycoerythrin-CF®594 tandem; PE-Cy5=Phycoerythrin-Cyanin 5 tandem; APC = Allophycocyanin; BV421= Brilliant Violet™ 421; Cartilage intermediate layer protein (CILP); Fibrinogen alpha chain (a fibrinogen); Fibrinogen beta chain (b fibrinogen).		

**Supplemental Table 3.** Antibodies, staining label and commercial source for phenotyping studies.			
**Marker**	**Color**	**Clone or Product**	**Vendor**
**Live/Dead**	FITC	LIVE/DEAD Fixable Green	ThermoFisher
**CD14**	FITC	HCD14	BioLegend
**CD19**	FITC	HIB19	BioLegend
**CD4**	V500	RPA-T4	BD
**CD45RA**	AF700	HI100	BD
**CCR7**	Cy5.5	G043H7	BioLegend
**CXCR3**	PE-Cy7	G025H7	BioLegend
**CCR4**	BV605	L291H4	BioLegend
**CCR6**	BV786	G034E3	BioLegend
**CD38**	BUV395	HB7	BD
**Abbreviations:** BD=BD Biosciences ; FITC=Fluorescein isothiocyanate; AF700= Alexa Fluor® 700; Cy5.5=Cyanin 5.5; PE-Cy7= PE-Cy5=Phyco-erythrin-Cyanin 7 tandem; BV605=Brilliant Violet™ 605; BV786=Brilliant Violet™ 786; BUV395= n Brilliant™ Ultraviolet polymer dye 395			
