# Supplementary material for: Multifaceted immune dysregulation characterizes individuals at-risk for rheumatoid arthritis
Source: Nat Commun. 2023 Nov 22;14:7637. doi: 10.1038/s41467-023-43091-8 (PMC10665556; doi:10.1038/s41467-023-43091-8)
Supplement: Supplementary file 2 — Description of Additional Supplementary Files [file 41467_2023_43091_MOESM2_ESM.pdf]

SUPPLEMENTAL DATA LEGENDS

Supplemental Data 1. Differentially methylated loci (DML) and differentially methylated genes (DMG) identified by comparison of anti-CCP3(-), At-Risk and Early RA in B cells, memory T cells and naïve T cells. Source data are provided as a Source Data file.

Supplemental Data 2. Union of top 10% most important differentially methylated loci (DML) selected by random forest models that separate anti-CCP3(-), At-Risk, and Early RA samples. After feature selection, the models achieved accuracies of 89.7%, 78.1% and 96.8% on the test data when discriminating between Early RA vs At-Risk, Early RA vs anti-CCP3(-) Controls and At-Risk vs anti-CCP3(-) Controls, respectively. A differentially methylated gene (DMG) is listed if a DML is identified in the gene's promoter region or gene body. Source data are provided as a Source Data file.

Supplemental Data 3. Antibody levels to individual or groups of similar antigens in pairwise comparisons between anti-CCP3(-) Controls and At-Risk, and At-Risk and Early RA. The levels of each antibody on the array are expressed as means of the fluorescence intensity. The p-values are pairwise comparisons from the analyses using linear regression and adjusting for age, sex and smoking status (ever/never) and include a p-value significant at <0.05, and in parentheses, a False Discovery Rate (FDR)-adjusted p-value where a value of <0.05 is considered significant (two-sided testing). Source data are provided as a Source Data file. Abbreviations: Cartilage intermediate layer protein (CILP); Fibrinogen alpha chain (fibrinogenA); Fibrinogen beta chain (fibrinogenB); Histone H4 (H4); Histone 2A (H2A); beta actin (B-actin); FDR=false discovery rate.

Supplemental Data 4. Antibody positivity in pairwise comparisons between anti-CCP3(-) Controls and At-Risk, and At-Risk and Early RA. The positivity of an antibody (or summed level) was determined as a level >=3 standard deviations above the mean level of all of the anti-CCP3(-) Control samples. The positivity of each autoantibody on the array are expressed as n (%) of samples that tested positive. The p-values are pairwise comparisons from the analyses using logistic regression for singular variables and Poisson regression for summed variables, and adjusted for age, sex and smoking status (ever/never) and include a p-value significant at <0.05, and in parentheses, a False Discovery Rate (FDR)-adjusted p-value where a value of <0.05 is considered significant (two-sided testing). Source data are provided as a Source Data file. Abbreviations: Cartilage intermediate layer protein (CILP); Fibrinogen alpha chain (fibrinogenA); Fibrinogen beta chain (fibrinogenB); Histone H4 (H4); Histone 2A (H2A); beta actin (B-actin); FDR=false discovery rate; SD=standard deviation.

Supplemental Data 5. Antibody levels to individual or groups of similar antigens in pairwise comparisons between anti-CCP3(-) Controls and At-Risk, and At-Risk and Early RA, with participants stratified by the presence/absence of the *0401 allele. The levels of each autoantibody on the array are expressed as means of the fluorescence intensity. The p-values are pairwise comparisons from the analyses using linear regression and adjusting for age, sex

and smoking status (ever/never) and include a p-value significant at <0.05, and in parentheses, a False Discovery Rate (FDR)-adjusted p-value where a value of <0.05 is considered significant (two-sided testing). Source data are provided as a Source Data file. Abbreviations: Cartilage intermediate layer protein (CILP); Fibrinogen alpha chain (fibrinogenA); Fibrinogen beta chain (fibrinogenB); Histone H4 (H4); Histone 2A (H2A); beta actin (B-actin); FDR=false discovery rate.

Supplemental Data 6. Antibody positivity in pairwise comparisons between anti-CCP3(-) Controls and At-Risk, and At-Risk and Early RA, with participants stratified by the presence/absence of the *0401 allele. The positivity of an antibody (or summed level) was determined as a level >=3 standard deviations above the mean level of all of the anti-CCP3(-) Control samples. The positivity of each autoantibody on the array is expressed as n (%) of samples that tested positive. The p-values are pairwise comparisons from the analyses using logistic regression for singular variables on Poisson regression for summed variables, and adjusting for age, sex and smoking status (ever/never) and include a p-value significant at <0.05, and in parentheses, a False Discovery Rate (FDR)-adjusted p-value where a value of <0.05 is considered significant (two-sided testing). Source data are provided as a Source Data file. Abbreviations: Cartilage intermediate layer protein (CILP); Fibrinogen alpha chain (fibrinogenA); Fibrinogen beta chain (fibrinogenB); Histone H4 (H4); Histone 2A (H2A); beta actin (B-actin); FDR=false discovery rate; SD=standard deviation
